# Pyrazinamide kills *Mycobacterium tuberculosis* via pH-driven weak-acid permeation and cytosolic acidification

**DOI:** 10.1101/2025.09.26.678883

**Published:** 2025-09-27

**Authors:** Janïs Laudouze, Tatyana I. Rokitskaya, Akira Abolet, Vanessa Point, Alexander M. Firsov, Ljudmila S. Khailova, Jean-François Cavalier, Stéphane Canaan, Alain R. Baulard, Yuri N. Antonenko, Alexandre Gouzy, Pierre Santucci

**Affiliations:** 1 Aix Marseille Univ, CNRS, LISM, IMM FR3479, IM2B, Marseille, France; 2 Belozersky Institute of Physico-Chemical Biology, Lomonosov Moscow State University, 119991 Moscow, Russia; 3 Univ. Lille, CNRS, Inserm, CHU Lille, Institut Pasteur de Lille, U1019 - UMR9017 - CIIL - Center for Infection and Immunity of Lille, F-59000 Lille, France.; 4 Department of Microbiology and Immunology, Weill Cornell Medical College, New York, New York, USA.

**Keywords:** acidic environments, acid-base equilibrium, intrabacterial pH homeostasis, protonophore, pantothenate, glycerol

## Abstract

Pyrazinamide (PZA) is a cornerstone drug in tuberculosis (TB) treatment with a strong bactericidal activity *in vivo* on both actively and non-replicating bacterial subpopulations. Yet the precise mode of action of its active form, pyrazinoic acid (HPOA), remains unclear.

In this study, we comprehensively explore and challenge the two major and conflicted models of PZA mode of action. The pH-dependent model, where the drug is mostly effective at acidic pH by acidifying *Mycobacterium tuberculosis* (*Mtb*) cytosol, and the PanD-dependent model where PZA’s active form targets the aspartate decarboxylase PanD, therefore depleting pantothenate and subsequently coenzyme A (CoA) levels regardless of the surrounding pH.

By combining standard antimicrobial susceptibility testing at various pH with fluorescence-based live recording of *Mtb* intrabacterial pH, we demonstrate that PZA kills *Mtb* by decreasing intrabacterial pH, independently of pantothenate levels. Comparative studies between a prototrophic *Mtb* strain and a pantothenate auxotrophic mutant lacking the *panCD* locus confirmed that PZA bactericidal activity is primarily driven by pH and its ability to acidify *Mtb* cytosol, independently of the aspartate decarboxylase PanD. Bio-electrophysiology experiments revealed that acidic pH promotes the conversion of the pyrazinoate anion POA^−^ into HPOA which in turn acts as conventional weak acid that facilitates membrane permeation and cytosolic acidification. Finally, using custom-based culture media, we demonstrate that PZA displays heterogeneous efficacy according to the media composition, therefore proposing a revisited biological model that might explain the discrepancies around PZA’s unique mode of action.

Overall, this work constitutes the first comprehensive side-by-side investigation of the two models and univocally supports a pH-dependent mechanism of action underlying PZA sterilizing activity, providing new insights for the development of more effective PZA-like drugs.

## Introduction

According the WHO’s Global Tuberculosis Report 2024, tuberculosis (TB) remains the leading cause of death worldwide due to an infectious agent with 1.25 million deaths and approximately 10.8 million new cases^1^. For drug-susceptible TB, WHO still recommends the long standing six-month, two-phase regimen that includes a daily cocktail of isoniazid (INH), rifampicin (RIF), ethambutol (EMB), and pyrazinamide (PZA) during 2-months followed by 4 months of RIF and INH. Globally, this regimen achieved a treatment-success rate of 88% for patients who started therapy in 2022, the most recent cohort with complete outcome data^1^. Despite its effectiveness, taking multiple drugs daily for six months often affects patient compliance which can ultimately lead to treatment failures and the emergence of drug-resistant strains^1^. Therefore, deepening our understanding of the mechanisms of action of existing anti-TB agents is essential to develop shorter, safer and more potent regimens that improve compliance and curb resistance^2^.

Among the four front-line anti-TB drugs, PZA holds a singular position. Whereas the molecular target of INH, RIF and EMB are now well-defined, PZA’s mode of action is still actively debated. This is likely due to the fact that PZA is the prototype of a context-dependent drug, that displays very potent efficacy *in vivo* but almost no antibacterial effect in standard mycobacterial broth cultures^3, 4, 5^. Nevertheless, according to the well-acknowledged Mitchison hypothesis^6^, its unique *in vivo* sterilizing activity, particularly against non-replicating tubercle bacilli, is what allows the standard of care six-month regimen to prevent relapses^6, 7^. In that context, PZA remains a key component of most first line current combinations^8^.

Discovered in 1952, the anti-TB properties of PZA were first reported following extensive synthesis of nicotinamide (NAM) analogues and the subsequent screening of their antibacterial activities within TB-mouse models of infection^9, 10^. Moreover, the high sterilizing properties of PZA when combined with INH and/or RIF, resulted in its inclusion in the standard anti-TB drug regimen^11, 12^.

Since its discovery in the 1950’s, seminal studies have demonstrated that PZA is a prodrug that requires to be bio-converted into pyrazinoic acid (HPOA) to be effective. This activation step is mediated by PncA, a non-essential *Mtb* pyrazinamidase/nicotinamidase involved in the nicotinamide adenine dinucleotide (NAD^+^) salvage pathway^13, 14^. Consistently, the majority of PZA-resistant clinical isolates harbour mutations in the promoter or the coding region of the gene *pncA* which reduce or abolish PncA production, stability or enzymatic activity, therefore preventing PZA bio-conversion^15, 16, 17, 18^.

Beyond this obligatory activation step, the downstream molecular events remain contentious. Early proposals, including inhibition of fatty acid synthetase I (FAS I) or the *trans*-translation machinery, did not survive experimental scrutiny^19, 20, 21, 22^. However, two competing models remain supported by distinct experimental evidence: the pH-dependent weak-acid permeation model and the PanD/CoA-inhibition model.

PZA’s antibacterial activity was originally proposed to be target-independent and driven by the acidic pH that surrounds *Mtb*^23, 24, 25, 26^. In this first model, PZA diffuses through the mycobacterial cell-wall, and is converted into the negatively charged pyrazinoate anion (POA^−^) by the cytoplasmic PncA enzyme. This negatively charged POA^−^ is further exported extracellularly through an active process that is not yet fully characterised. Then, the protonation state of the HPOA/POA^−^ acid-base pair in the vicinity of the bacterium is dictated by the Henderson-Hasselbalch equation, which relates the pKa of the carboxylic acid function (pKa = 3.6) to the surrounding extrabacterial pH^24, 26, 27, 28^. Accordingly, highly acidic environment drives the equilibrium towards the neutral protonated HPOA form, which efficiently diffuses through the mycobacterial cell wall and further dissociates into POA^−^ and H^+^ when reaching the circum-neutral bacterial cytosolic pH. The release of protons inside *Mtb* cytoplasm causes a decrease in the intrabacterial pH (IBpH) and membrane potential which has been reported to be critical for PZA antimicrobial activity^24, 26, 28, 29, 30^.

Nonetheless, this model has been actively challenged over the past decades and an alternative, pH-independent, mode of action has been proposed^31, 32, 33, 34, 35, 36, 37^. Genomic analysis of *in-vitro* selected *Mtb* low-level resistant mutants to POA^−^/HPOA revealed mutations in distinct genes including the *panD* gene, suggesting that the aspartate decarboxylase PanD, involved in Coenzyme A (CoA) synthesis, could be the primary target of the drug^31, 32, 33, 38^. Further biochemical studies have proposed that POA^−^ could inhibit PanD activity by binding to its active site^36^, or alternatively triggering its degradation by the ClpC1-ClpP proteolytic system^37^. Hence, according to this second model the cytoplasmic bioactivated POA^−^ anion binds to PanD, limits its activity and induces its degradation, thus ultimately preventing CoA synthesis independently of the extrabacterial pH^36, 37^.

Given the ongoing debate surrounding the mode of action of PZA, we aimed at comprehensively challenge these two models side-by-side. By combining standard antimicrobial susceptibility testing at various pH with fluorescence-based live recording of *Mtb* IBpH and bio-electrophysiology experiments, we demonstrate that PZA/HPOA alters cytosolic pH of both actively-growing and non-replicating *Mtb* through a weak acid permeation model. Our study reports that this process was only minimally affected by pantothenate levels and genetic studies, using a *panCD* mutant strain, confirmed that PZA bactericidal action is primarily driven by acidic pH and its ability to acidify the *Mtb* cytosol, independently of the aspartate decarboxylase PanD. Finally, by using custom-designed culture media containing physiologically relevant lipid-based carbon sources, we demonstrate that the antagonistic effect of the CoA precursors observed in standard 7H9 media are no longer detectable. These results not only shed new light on the mode of action of PZA but also explain some of the long-standing discrepancies between its observed *in vitro* and *in vivo* activities.

## Results

### Comprehensive pH-dependent analysis of PZA/HPOA antibacterial activity

**Figure 1.**
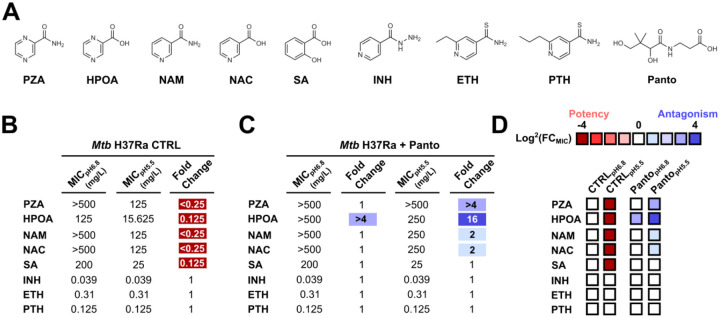
Comprehensive analysis of PZA/HPOA antibacterial activity against *Mtb*. **(A)** Structure of the compounds investigated in this study. **(B)** *Mtb* antibiotic susceptibility profiling performed at circum-neutral and acidic pH in the absence or **(C)** presence of Panto antagonists. Results are expressed as minimal inhibitory concentration (MIC). **(D)** Potency and antagonisms are further displayed as color-coded heat map from red (potency) to blue (antagonism). Heat map intensities for each compound represent the fold change (FC) in MIC values expressed as Log^2^(FC_MIC_) by applying the following formula MIC_pH5.5_/MIC_pH6.8_ or MIC_Antagonist_/MIC_CTRL_. All results are representative of three independent technical replicates performed on three independent occasions.

Since the pH-dependent mode of action of PZA/HPOA has recently been challenged, we first aimed to re-evaluate the impact of mildly acidic pH on the efficacy of a panel of anti-TB drugs including PZA/HPOA (Fig 1A). Antimicrobial susceptibility testing was then performed in standard 7H9 Middlebrook medium at pH 6.8 and pH 5.5, and the minimal inhibitory concentration (MIC) against *Mtb* H37Ra was determined for each compound (Fig 1B). As expected, PZA was inactive when assessed at pH 6.8 with MIC values exceeding 500 mg/L, while HPOA displayed a very moderated activity with an MIC of 125 mg/L. As previously reported^24, 25^, both PZA and HPOA exhibited significant pH-dependent growth inhibition patterns with an approximate 4 to 8-fold decrease in MIC values when tested at pH 5.5 (Fig 1B). This pH-dependent effect was also easily visualised in a heatmap using color-coded representation based on Log^2^(FC_MIC_) values (Fig 1D). Additional experiments with the natural substrate and product of PncA, nicotinamide (NAM) and nicotinic acid (NAC) respectively, showed similar inhibition patterns with at least a 4-fold gain in potency when tested at acidic pH, thereby reflecting a pH-dependent activity (Fig 1B). As previously observed, salicylic acid (SA), used in control experiment, showed a strong potency at acidic pH with a significant shift from 200 mg/L to 25 mg/L^39, 40^. By contrast, no variation in MIC at both pH levels was observed with isoniazid (INH), ethionamide (ETH) and prothionamide (PTH), three well-established prodrugs that target the 2-trans-enoyl-ACP reductase InhA (Fig 1B). This confirms that the observed pH-dependent activity is compound and/or mode of action dependent rather than universal. Altogether, these results support a model in which an acidic pH environment potentiates the activity of several anti-TB drugs or prodrugs bearing carboxylic acid function, conferring weak acid properties, including PZA and HPOA.

### Supplementation with CoA precursors involved downstream of the aspartate decarboxylase PanD antagonise PZA/HPOA efficacy in standard Middlebrook 7H9.

The aspartate decarboxylase PanD catalyses the formation of β-alanine (β-ala) from L-aspartate by removing a carboxyl group through the consumption of a proton H^+^ and generation of CO_2_. Condensation of the β-ala with one pantoate molecule is performed by the pantothenate synthetase PanC in an ATP-dependent reaction to produce pantothenate (Panto), which is essential for building up CoA through the action of the CoaABCDE proteins (S1 Fig). One of the main evidences supporting PanD as the primary target of PZA/HPOA was based on the observation that supplementation of complete 7H9 Middlebrook medium with CoA-biosynthesis precursors, such as Panto, negatively impacted the efficacy of PZA/HPOA at pH 6.8 and pH 5.5^32, 41^. To confirm these results, we performed a comprehensive analysis of *Mtb*-growth inhibition profiles with our subset of drugs at both circumneutral or mildly acidic pH in the presence of exogenous Panto. In good agreement with literature data, the presence of 25 mg/L of Panto strongly antagonised PZA/HPOA efficacy at both pH, resulting in an increase in their MIC values from 4- to 8-fold (Fig 1C & Fig 1D). Interestingly, this modulator only displayed a very weak antagonistic effect at acidic pH against NAM/NAC (Fig 1C & Fig 1D), in line with previous observations^41^. Finally, no impact on the anti-TB drugs INH, ETH and PTH was observed, nor with the weak acid control molecule SA (Fig 1C & Fig 1D). All these results confirm the multiple observations from independent groups, supporting the concept that supplementation with Panto specifically antagonise PZA/HPOA efficacy in complete 7H9 Middlebrook media.

### *panCD* locus and CoA-precursors supplementation have minimal effect on PZA/HPOA-induced intrabacterial pH disruption on replicating *Mtb*.

We and others have previously proposed that PZA/HPOA efficacy was directly linked to its ability to alter *Mtb* intrabacterial pH homeostasis (IBpH) in a pH-dependent manner^24, 29, 30^. To confirm these results, we capitalized from a well-established *Mtb* reporter strain that produces a ratiometric pH-GFP sensor, enabling the non-invasive monitoring of IBpH changes during drug treatment (Fig S2)^29, 30, 39, 42, 43^. Using this genetic tool, we set up an *in vitro* assay and calibrated the response of this fluorophore using lysates from recombinant *Mtb* H37Ra pH-GFP reporter strain at different pH increments (Fig S2A; top panel). Locally estimated scatterplot smoothing (LOESS) curve fitting was performed in order to obtain a reliable standard curve that links *Mtb* pH-GFP fluorescence ratio to IBpH values (Fig S2A; bottom panel). To validate that this experimental system fully enables the quantitative monitoring of compound-mediated IBpH perturbation *in vitro*, the protonophore carbonyl cyanide *m*-chlorophenyl hydrazone (CCCP) and the pH-dependent ionophore monensin (MON) were used as positive controls (Fig S2B). Exponentially growing *Mtb* pH-GFP was pulsed for 24 h in complete 7H9 Middlebrook medium with increasing concentrations of CCCP (Fig S2B; top panel) or MON (Fig S2A; bottom panel) at circumneutral and acidic pH, respectively. Results showed that both compounds sharply decreased *Mtb* pH-GFP ratio in a dose-dependent manner until reaching IBpH values lower than IBpH 5.5, therefore validating our experimental approach. Control experiments were also performed at pH 6.8 or pH 5.5 in complete 7H9 media in presence/absence of high exogenous Panto levels (*e.g*., 25 mg/L) and showed no major changes (*e.g*., pH-GFP ratios values in between 0.8 and 0.9 in all conditions tested), approximating a constant IBpH value of IBpH 6.5, in line with previous reports^29, 30, 39, 42, 43^ (Fig S3A).

**Figure 2.**
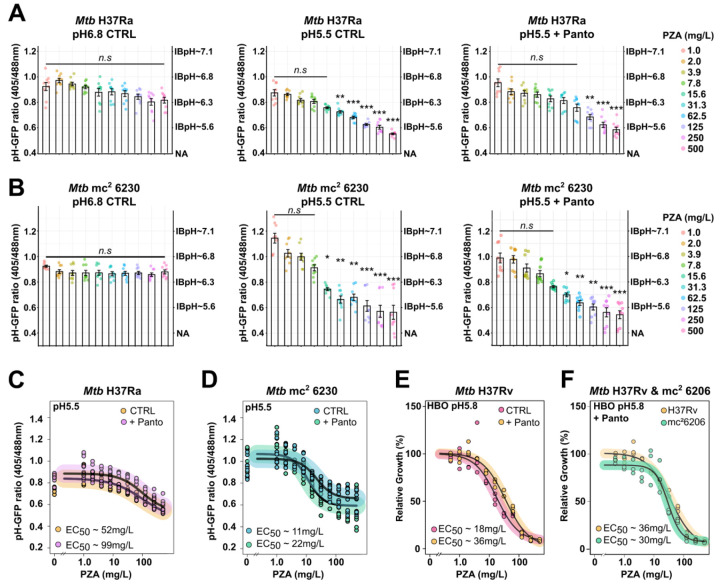
PZA/HPOA displays pH-dependent antibacterial activity on actively growing *Mtb* by primarily impacting IBpH homeostasis. **(A)** Quantification of PZA-induced *Mtb* H37Ra IBpH alteration at circum-neutral (*left* panel), acidic pH (*middle* panel) and acidic pH in the presence of 25 mg/L of Panto (*right* panel). *Mtb* pH‐GFP ratio were determined in the presence of increasing concentrations of PZA after 24 h of exposure. **(B)** Quantification of PZA-induced *Mtb* mc^2^ 6230 IBpH alteration at circum-neutral (*left* panel), acidic pH (*middle* panel) and acidic pH in the presence of 25 mg/L of Panto (*right* panel). *Mtb* pH-GFP ratio were determined in the presence of increasing concentrations of PZA after 24 h of exposure. **(C)** Comparative analysis of PZA-mediated IBpH alteration against *Mtb* H37Ra in the absence or presence of Panto. Dose-response analysis displayed were obtained following a 4-parameter nonlinear logistic regression of the data displayed in the middle and right panels of Fig 2A, and EC_50_ were determined accordingly. **(D)** Comparative analysis of PZA-mediated IBpH alteration against *Mtb* mc^2^ 6230 in the absence or presence of Panto. Dose-response analysis displayed were obtained following a 4-parameter nonlinear logistic regression of the data displayed in the middle and right panels of Fig 2B, and EC_50_ were determined accordingly. *Mtb* IBpH results displayed in this figure were obtained from 3 biologically independent experiments and are displayed as mean ± SEM. In dose response analysis, statistical significance was assessed by comparing the means of each concentration with the lowest concentration tested using one-way ANOVA followed with Tukey’s multiple comparisons test. All *p*-values were considered significant when *p*-value < 0.05. **(E)** Comparative analysis of PZA-mediated growth inhibition against *Mtb* H37Rv in HBO medium pH 5.8 and in the absence (pink) or presence of Panto (yellow) antagonist. Dose-response analysis displayed were obtained following a 4-parameter nonlinear logistic regression and EC_50_ were determined accordingly. Results are representative of three independent replicates. **(F)** Comparative analysis of PZA-mediated growth inhibition against *Mtb* H37Rv WT (yellow) and *Mtb* H37Rv mc^2^ 6206 (green) in HBO medium pH 5.8 in the presence of 25 mg/L Panto antagonist. Dose-response analysis displayed were obtained following a 4-parameter nonlinear logistic regression and EC_50_ were determined accordingly. Results are representative of three independent replicates.

Dose response analysis upon PZA treatment confirmed previous observations that reported no major cytosolic acidification when assessed at circumneutral pH (Fig 2A; left panel). Conversely, at pH 5.5 PZA strongly impairs *Mtb* IBpH homeostasis, supporting a pH-dependent effect (Fig 2A; central panel). Importantly, these changes were deemed of statistical significance from 31.3 mg/L, a concentration that is commonly observed in plasma and lungs of TB patients undergoing chemotherapy^44, 45, 46^. Similar results were obtained regarding *Mtb* IBpH when treated with the active form HPOA but only at high concentration (Fig S3B). This could be explained by its poor solubility and increased binding affinity to bovine serum albumin contained in the medium in comparison to PZA^25^. Control experiments with INH did not show any effect on *Mtb* IBpH, whatever the pH and the tested concentrations (Fig S3C). These results, which agree with previous reports^29, 30^, show that *Mtb* growth arrest mediated by high concentrations of antibiotic at mildly acidic pH level does not necessarily result from IBpH disruption^39^; but also confirm that IBpH perturbation is only observed with a specific subset of drugs, including PZA/HPOA.

In order to establish whether the recorded IBpH homeostasis perturbations were linked to the PanD-dependent mode of action, and therefore CoA-biosynthesis defect, the same subset of experiments was repeated in the presence of 25 mg/L Panto (Fig 2A; right panel), a high exogenous concentration known to fully rescue the growth of an auxotrophic *Mtb panCD* mutant (i.e., *Mtb* mc^2^ 6230 strain)^47, 48^. In this context, Panto supplementation had a mild but detectable and statistically significant effect on PZA or HPOA-mediated IBpH perturbation (Fig 2A; right panel & Fig S3B). Indeed, in the presence of Panto, 125 mg/L of PZA where required to trigger significant IBpH acidification, as compared to 31.3 mg/L in its absence. This result is in line with the negative effect of Panto on PZA/HPOA efficacy observed during antimicrobial susceptibility profiling assays.

To go further, we decided to complement this series of experiments by relying on a genetic approach. Hence, we tested whether *panCD* deletion could interfere with PZA/HPOA-mediated IBpH homeostasis perturbation. We first confirmed that the *Mtb* mc^2^ 6230 strain was auxotroph for Panto in our experimental system (Fig S4A). Then, we introduced the pUV15-pHGFP vector in this genetic background to generate a *Mtb* mc^2^ 6230 pH-GFP reporter strain. As for its prototrophic counterpart, pH-GFP ratios remained constant between 0.85 and 1.0, both at pH 6.8 or pH 5.5 and in the presence or absence of Panto (Fig S4B). The fact that this strain was perfectly able to maintain its cytosolic pH around IBpH 6.5–6.8 suggests that CoA auxotrophy might not trigger major IBpH maintenance defects within the first 24 h of depletion.

Dose-response analysis of PZA/HPOA impact on IBpH homeostasis using the exact same experimental setting as presented above showed that no major changes in *Mtb* mc^2^ 6230 IBpH when assessing PZA or its active counterpart HPOA at circumneutral pH (Fig 2B; left panel & S3C Fig). Interestingly, at pH 5.5, a statistically significant change in IBpH acidification caused by PZA was observed starting from 15.6 mg/L (Fig 2B; central panel). Finally, experiments performed in the presence of 25 mg/L of Panto showed a very minor antagonistic effect on PZA/HPOA-mediated acidification (Fig 2B; right panel & Fig S4C).

To better characterise the role of Panto and *panCD* locus in PZA/HPOA mode of action, we performed a log-logistic regression analysis of the pH-GFP ratios obtained in Fig 2A & Fig 2B and estimated the half maximal effective concentration (EC_50_) of PZA required to alter *Mtb* IBpH (Fig 2C & Fig 2D). Both *Mtb* H37Ra and *Mtb* mc^2^ 6230 *panCD* mutant exhibited similar dose-response curves, with a two-fold increase in the EC_50_ of PZA in the presence of Panto. Moreover, the results also showed that a *panCD* auxotroph mutant is slightly more sensitive to PZA-mediated IBpH disruption than a prototroph strain (EC_50_ 11 mg/L *vs*. 50 mg/L for *Mtb* H37Ra vs. *Mtb* mc^2^ 6230, respectively), and that supplementation with 25 mg/L Panto interferes only very modestly with PZA-mediated IBpH disruption in the two genetic backgrounds (EC_50_ 22 mg/L and 99 mg/L, respectively). Accordingly, these results suggest that the enzymatic reactions carried out by PanCD and/or their down-products might help to mitigate PZA-triggered IBpH acidification, but only at relatively low concentrations of PZA.

Altogether, our data reveal that, even though the deletion of *panCD* and Panto supplementation alter PZA-mediated IBpH disruption to some extent, their impact is minor. Indeed, the disruption of IBpH homeostasis does not appear to depend directly on PanD function or its downstream CoA biosynthetic pathway. Instead, these IBpH alterations occur primarily in a pH-dependent manner and are independent of global CoA deficiency or availability of its precursors. Therefore, our data strongly indicate that PanD is not the primary target of PZA/HPOA.

### Media composition modulates CoA precursors antagonism on PZA/HPOA efficacy against *Mtb*.

It is well acknowledged that media composition, including carbon-sources, can significantly impact the efficacy of antibiotics *in vitro*^49, 50^. With regards to PZA/HPOA, Gopal *et al*. showed that an increasing concentration of glycerol *in vitro* hypersensitises *Mtb* to HPOA further highlighting that the nature but also the availability of carbon source might alter drug efficacy^33^. To confirm this observation, we first compared *Mtb* H37Ra susceptibility profile towards HPOA at mildly acidic pH 5.5 in 7H9 standard media containing 10% OADC in presence or absence of 0.2% glycerol (Fig S5). The obtained results confirm that the presence of this non-physiological concentration of glycerol^51^ enhances HPOA efficacy, with EC_50_ values shifting from 38 mg/L to 9 mg/L and MIC values from 500 mg/L to 15.6 mg/L in glycerol-containing media (Fig S5A–B). Similar significant changes in MIC values, shifting from >500 mg/L to 125 mg/L, were also observed with PZA in the presence of glycerol; whereas no alteration in MIC values was reached with the control drug SA (Fig S5C). Remarkably, the presence of glycerol significantly increases PZA/HPOA efficacy (Fig S5A–B). From these findings, we further investigated how carbon sources alter PZA/HPOA efficacy. Since the 7H9 Middlebrook media supplemented with OADC contains both glycolytic and lipid carbon sources, we opted for custom-made minimal media of perfectly controlled and known composition. We capitalized from the recently developed High-BSA-Oleate (HBO) medium, that rely on high-BSA content and oleic acid a sole carbon source to assess PZA activity^52^. Using this experimental system at pH 5.8, we confirmed that *Mtb* H37Rv reference strain was not only susceptible to PZA (EC_50_ ~18 mg/L), but also that the negative effect of Panto was almost completely lost, with EC_50_ ~36 mg/L, respectively (Fig 2E). Since Panto had a very limited effect on PZA efficacy in this custom media, we took advantage of this phenotype and perform susceptibility profiling of a *Mtb* H37Rv mutant strain devoid on *panCD* locus: *Mtb* mc^2^ 6206 (Fig 2F). Comparative analysis of the susceptibility profile of WT and *panCD* mutant in presence of Panto showed no major differences, with EC_50_ values of 36 mg/L and 30 mg/L, respectively. In this more physiological medium, our results clearly demonstrate that PZA efficacy is independent of Panto supplementation or the presence of a functional *panCD* locus.

### PZA/HPOA-mediated pH-disruptive effect against non-replicating *Mtb* is driven by the availability of surrounding protons.

According to the Mitchison hypothesis, the sterilizing activity of PZA is greatly enhanced against non-replicating bacteria^27, 53^. This was confirmed by independent groups that reported potent bactericidal activity *in vitro* when assessed on starved non-replicating *Mtb*^25, 35, 54^. To go further in the characterisation of PZA’s mode of action, we investigated PZA-mediated IBpH disruption within the well-established phosphate-citrate buffer (PCB). This minimalist experimental system enables to test compound-mediated IBpH homeostasis perturbation, at different pH increments on non-replicating starved bacteria^29, 42^. Hence, we first monitored *Mtb* H37Ra adaptation to PCB after 48 h of incubation at different pH ranging from pH 5.5 to pH 7 (Fig S6). Monitoring of IBpH revealed that, as pH decreases, *Mtb* H37Ra slightly equilibrates its IBpH towards more alkaline values. This phenomenon is likely a compensatory mechanism in response to protons influx and changes in membrane potential^55^. Interestingly, such pH-dependent adaptation in IBpH was not detectable in standard supplemented Middlebrook 7H9 medium (Fig S3A), suggesting that IBpH homeostasis might be modulated by multiple exogenous factors including nutrients availability during pH variations.

**Figure 3.**
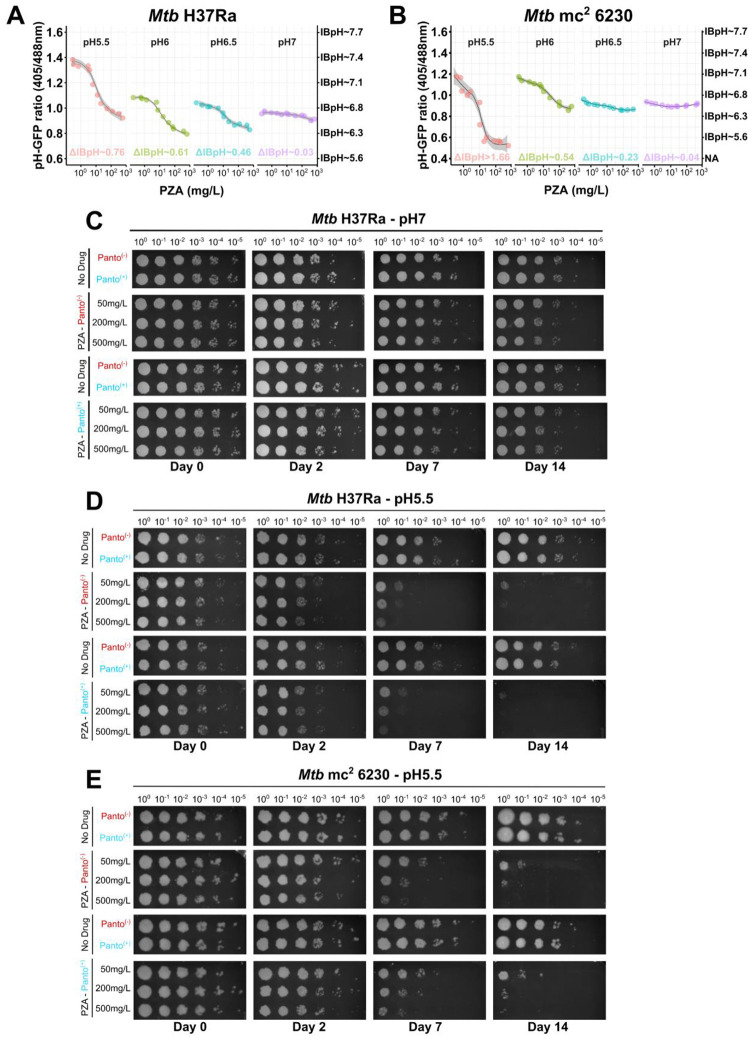
Proton availability dictates the ability of PZA/HPOA to acidify the cytosol and kill non-replicating *Mtb* independently of the *panCD* locus and pantothenate levels. **(A-B)** pH-dependent PZA-mediated IBpH cytosolic acidification of *Mtb* H37Ra (**A** – *left* panel) and *Mtb* mc^2^ 6230 (**B** – *right* panel). Dose-response analysis were performed in PCB buffer adjusted at pH 7 (purple), pH 6.5 (blue), pH 6 (green) and pH 5.5 (red). *Mtb* were exposed to increasing concentrations of PZA from 0.25 to 500 mg/L for 48 h before pH-GFP ratio recording. Using pH-GFP ratios, distinct models were built using the LOESS function and ΔIBpH were calculated by comparing the IBpH recorded at the highest PZA concentration tested with the one at the lowest PZA concentration included in the dose response for each tested pH. **(C-D-E)** pH-dependent bactericidal activity of PZA on non-replicating *Mtb* H37Ra and *Mtb* mc^2^ 6230 pH-GFP cells. Survival in PCB buffer was performed by pulsing 1 × 10^7^ *Mtb* CFU/mL with increasing concentration of PZA including 0 mg/L, 50 mg/L, 200 mg/L and 500 mg/L in the presence or absence of 25 mg/L Panto. At day 0, 2, 7 and 14, *Mtb* cells were recovered, serially diluted and spotted onto 7H11 agar media in the presence 25 mg/L Panto and 50 mg/L hygromycin B. Experiments were performed with *Mtb* H37Ra at pH 7 (**C** – *top* panel) and at pH 5.5 (D – *middle* panel), and with *Mtb* mc^2^ 6230 at pH 5.5 (**E** – *bottom* panel). Results are representative of two distinct experiments performed on independent occasion.

Such approach was further used to monitor the effect of several drugs on *Mtb* H37Ra IBpH homeostasis at various pH, with RIF, and MON as negative and positive controls respectively (Fig S7A–B). First, dose response analysis of PZA treatment showed a clear dose-dependent and pH-dependent effect on *Mtb* H37Ra IBpH (Fig 3A). Similar results were also obtained with NAM/NAC (Fig S7C–D) and HPOA (Fig S8A) in agreement with their previously observed pH-dependent potency. Additional control experiments with PTH, ETH and INH (Fig S7E–G) confirmed once again that such process was specific to PZA/HPOA and its analogues.

When carried out with the *Mtb* mc^2^ 6230 mutant strain, results showed that the lack of aspartate decarboxylase PanD had only a minimal impact on the pH-dependent IBpH-disruptive effect of PZA (Fig 3B) or HPOA (Fig S8B) in comparison to its prototrophic *Mtb* H37Ra counterpart. We also noticed that with PZA/HPOA the global decrease in IBpH was greater for the *Mtb* mc^2^ 6230 mutant than the WT, with ΔIBpH >1.66/>1.42 and 0.76/0.94 pH units, respectively.

Determination of PZA’s EC_50_ in this experimental system at pH 5.5, on both *Mtb* H37Ra and *Mtb* mc^2^ 6230 mutant using log-logistic regression model, resulted in identical values of 11 mg/L and 10 mg/L between the two genetic backgrounds H37Ra and *panCD*, respectively (Fig S8C). Surprisingly, these values perfectly match the one obtained in 7H9 Middlebrook media for *Mtb* mc^2^ 6230 mutant (Fig 2D).

Taken together, these data confirm that PZA/HPOA can disrupt intrabacterial pH in non-replicating *Mtb* in a strictly pH-dependent manner, independent of PanD or CoA biosynthesis. While the absence of PanCD does not affect the determined EC_50_ for IBpH disruption, it results in greater pH collapse, suggesting a reduced buffering capacity under stress. These findings reinforce the idea that the availability of external protons is the critical driver of PZA/HPOA-mediated pH disruption in non-replicating bacilli.

### Bactericidal activity of PZA on non-replicating *Mtb* is independent of *panCD* locus or pantothenate level.

Since both prototrophic and auxotrophic strains display similar non-replicating behaviour in PCB system, and that PZA treatment at pH 5.5 resulted in important cytosolic acidification for both strains, we investigated whether such acidification could result in bacterial cell death independently of the *panCD* locus.

To do so, exponentially growing *Mtb* was washed several times before being normalized and incubated in PCB at pH 7 or pH 5.5 in the absence or the presence of increasing concentrations of PZA. Samples were collected at day 0, 2, 7 and 14, serially diluted and plated on complete agar media to perform CFU-based assessment of bacterial viability via spot assays (Fig 3C–E). A control experiment performed at pH 7 showed that PZA treatment, in the presence or absence of Panto, had no effect on *Mtb* H37Ra viability over the 14-days period even at high PZA concentration of 500 mg/L, confirming once again that PZA-mediated lethality is pH-dependent (Fig 3C).

Subsequently, experiments were performed at mildly acidic pH 5.5. A drug-free control condition was included as reference baseline and demonstrated that *Mtb* viability was not altered during the entire course of the experiment at pH 5.5. Supplementation of 25 mg/L of Panto had also no impact on *Mtb* H37Ra viability over the 14-day period. On the other hand, PZA treatment showed, as expected, a time and dose dependent effect on bacterial viability. Indeed, although very little effect on *Mtb* H37Ra survival was observed at day 2, treatment with 50, 250 or 500 mg/L PZA induced approximately 3-log^10^ reduction in bacterial viability at day 7. Finally, this bactericidal effect was exacerbated when the treatment lasted 14 days with an almost complete sterilization of the culture (Fig 3D). Supplementation of the PCB with 25 mg/L Panto had no effect on PZA-mediated cell death, suggesting that PZA sterilizing activity is completely independent of pantothenate level.

Similarly, analysis of *Mtb* mc^2^ 6230 survival in PZA-free condition revealed that CoA deprivation does not alter viability over the 14-day period, regardless of Panto supplementation (Fig 3E), in agreement with previous report^56^. In the presence of PZA, the *panCD* mutant strain exhibited a vulnerability pattern similar to the WT, showing noticeable time- and dose-dependent effects which resulted in an almost complete sterilization of the culture, independently of the presence of Panto.

Altogether, these data univocally support a pH-dependent bactericidal mode of action of PZA that does not require the targeting of PanD nor the disruption of the CoA biosynthesis pathway.

### Bio-electrophysiology experiments reveal that HPOA pH-disruptive activity is mediated by a weak acid permeation model.

PZA/HPOA-mediated alterations of IBpH homeostasis could be due to two major modes of action. In the first one, HPOA acts as a weak acid, diffusing its protons unilaterally across the cell-envelope and releasing them depending on the cytosolic pH and its pKa. On the other hand, HPOA exhibits protonophore-like properties and flip protons across the cell-membrane through repeated cycles.

**Figure 4.**
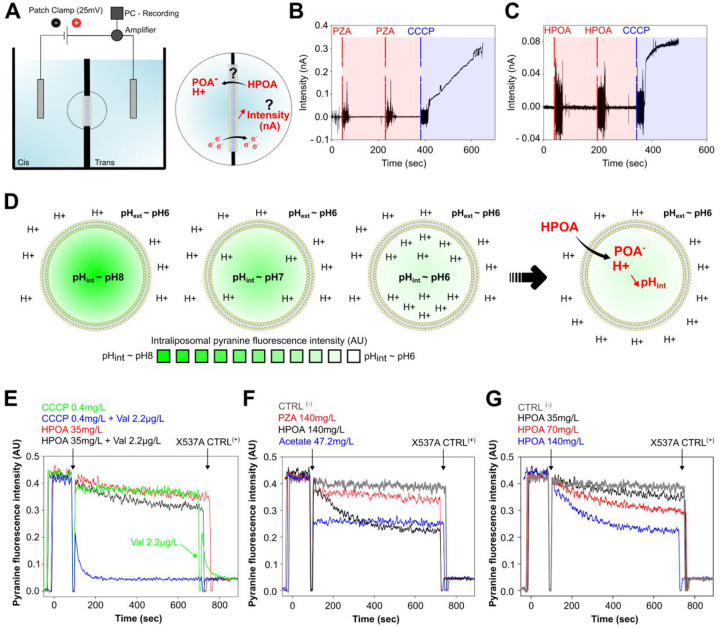
Bio-electrophysiology experiments support a weak acid model of membrane permeation and acidification by the active form HPOA. **(A)** Schematic representation of the BLM (made from DPhPC) electrophysiological setup used to test the protonophoric activity of PZA and HPOA. **(B-C)** PZA (panel **B**) or HPOA (panel **C**) does not induce electrical current through planar BLM made from DPhPC. The well-established protonophore CCCP was used as positive control. Experiments were carried out in a solution composed of 10 mM Tris, 10 mM MES, 100 mM KCl, pH 7.0. BLM voltage was set constant at 50 mV. **(D)** Schematic representation of the pyranine-loaded liposomes setup used to test the protonophoric activity and weak acid properties activity of PZA and HPOA. **(E)** Dissipation of the pH gradient by HPOA and CCCP on membranes of pyranine-loaded liposomes formed from mixed POPC, POPG and cholesterol. The black arrow at t_90s_ marks the addition of HPOA or CCCP. The kinetics of pyranine fluorescence intensity inside liposomes are shown for CCCP (0.4 mg/L, green curve), CCCP and valinomycin (Val) (0.4 mg/L and 2.2 μg/L, blue curve), HPOA (35 mg/L, red curve), and HPOA and valinomycin (35 mg/L and 2.2 μg/L, black curve). After t_600s_, the ionophore Lasalocide A (X537A) was added to the cuvette to equilibrate the pH inside and outside the liposomes. The lipid concentration in the cuvette was 20 mg/L. **(F)** Dissipation of the pH gradient by PZA or HPOA on membranes of pyranine-loaded liposomes, the time of addition is marked by the arrow at t_90s_. The kinetics of pyranine fluorescence inside liposomes are shown for HPOA (140 mg/L, black curve), PZA (140 mg/L, red curve), acetate (47.2 mg/L, blue curve). Grey represents the control condition with vehicle only (DMSO). **(G)** Dose-dependent effect of HPOA on pH-gradient dissipation on membranes of pyranine-loaded liposomes. Increasing concentrations of HPOA (35 mg/L, black curve), (70 mg/L, red curve), (140 mg/L, blue curve) were tested. After t_600s_, the control ionophore Lasalocide A (X537A) was added to the cuvette to equilibrate the pH inside and outside the liposomes. The lipid concentration in the cuvette was 20 mg/L.

In this context, we first examined the protonophoric activity of PZA and its active form HPOA in a pure bilayer lipid membrane (BLM) system and further measured the electric current across a planar lipid bilayer under voltage-clamp conditions (Fig 4A). As shown in Fig 4B–C, neither PZA nor HPOA induced a transmembrane electric current on BLM when assessed at pH 7. Subsequent addition of the well-established protonophore CCCP led to the induction of the BLM current, as expected for a protonophore in this experimental system. Therefore, our results suggest that PZA and HPOA are not able to induce electrical current in a pure system of planar bilayers.

Then, we aimed at confirming these results in a system made of pure liposomes which is complementary to the BLM. The cyclic movement of a protonophore through the lipid membrane, which results in a proton transport, is accompanied by a stage of an electrical charge transfer. Hence, in a system of pure liposomes, the generated diffusion potential on the membrane quickly blocks proton transport outside of the vesicles. Consequently, the study of protonophores requires the presence of an electrogenic ion carrier, such as valinomycin which is an electrogenic K^+^ carrier^57, 58^. So, to confirm our BLM results, we investigated the effect of PZA and HPOA on the intraliposomal pH-sensitive dye pyranine (Fig 4D) by monitoring its fluorescence intensity profiles in the presence (Fig 4E; black curve) and in the absence (Fig 4E; red curve) of valinomycin. The effect of HPOA-induced acidification was minor and almost not affected by the addition of valinomycin. In contrast, the effect of the well-established protonophore CCCP was very pronounced in the presence of valinomycin (Fig 4E; blue curve) and almost absent without this electrogenic K^+^ carrier (Fig 4E; green curve). This subset of experiment obtained within the pyranine-loaded liposomes system fully supports our previous BLM results and strongly argues against the protonophoric action of PZA and HPOA.

Alternatively, we also tested the ability of these compounds to impact the respiratory electron transport chains, which is another well-known characteristic of protonophores. The respiration rate of isolated mitochondria was determined by recording the activity of respiratory electron transport chains, which, in turn, is limited by the proton electrochemical gradient. Under the conditions of upper proton motive force limitation, the respiration rate is minimal and depends on the intrinsic proton leakage across the inner mitochondrial membrane. The addition of a protonophore diminishes the proton motive force value and stimulates respiration. In experiments measuring rat liver mitochondrial respiration, both PZA and HPOA poorly stimulated the respiration, while the addition of 2,4-dinitrophenol (DNP), a known protonophore, drastically stimulated the respiration (Fig S9). This suggests once again that the latter allows the cyclic translocation of protons, while PZA/HPOA did not.

We capitalized from the pyranine loaded liposome system to test whether PZA/HPOA have the ability to translocate protons and acidify the bacterial cytosol following a weak acid permeation model. According to this model, the protonation of weak acids into their neutral form increases their ability to cross biological membranes^59, 60, 61^, thereby raising their concentrations until they reach a steady-state level. More importantly, when protonated, these molecules unilaterally translocate through the membrane by passive diffusion and carry protons that, based on the pKa of the carboxylic acid function, might be subsequently released^25, 59^. To test this hypothesis, we treated pyranine-loaded liposomes with PZA or HPOA (Fig 4F). The well-characterised weak acid acetate, used as positive control, quickly acidified the content of the lumen (Fig 4F; blue curve). If PZA had no effect on intraliposomal pH, HPOA on the other hand was able to acidify the liposomal lumen (Fig 4F; red and black curves, respectively). Finally, increasing concentration of HPOA resulted in a dose dependent acidification of the lumen (Fig 4G), therefore fully supporting a conventional weak acid permeation model.

**Figure 5.**
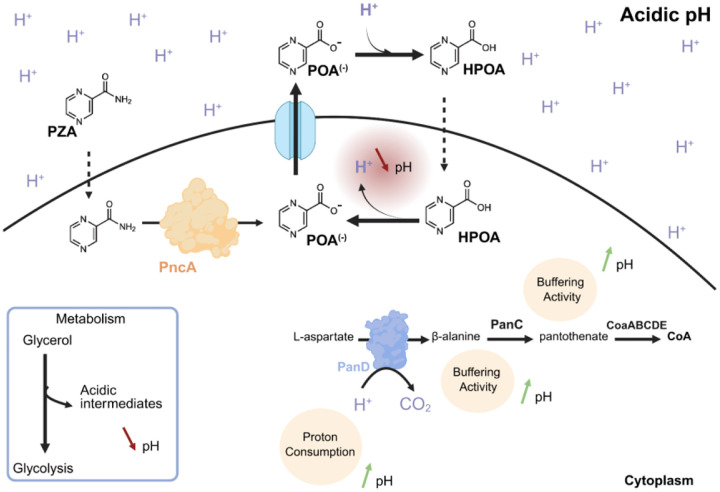
PZA/HPOA is bactericidal through pH-dependent weak acid permeation and cytosolic acidification as primary mechanism of action. Revisited model of PZA/HPOA efficacy, supporting a pH-dependent mechanism of weak acid permeation and cytosolic acidification as main mechanism of bactericidal activity. In this model, we propose that the presence of glycerol potentiates PZA/HPOA efficacy by generating acidic intermediates that lower IBpH. Accordingly, β-alanine and pantothenate might mitigate this effect by acting as buffering molecules that alleviate acidosis. Finally, PanD enzymatic activity consumes protons which in turn increases IBpH. Therefore, in this model PanD minimally contributes to PZA tolerance and cannot be considered as the primary target of the drug.

In summary, we propose a unifying model in which acidic pH, and not PanD inhibition, is the primary driver of PZA efficacy against *Mtb* through a weak acid permeation model (Fig 5). In this model, HPOA behaves as a membrane-permeant weak acid that enters in its protonated form and releases protons intracellularly, collapsing pH homeostasis in a pH-dependent manner. PanD enzymatic activity and exogenous supplementation with CoA precursors only modestly buffer this acidification process and only under less-physiological, glycerol-based *in vitro* conditions. This provide a molecular mechanistic explanation for why the previously observed PanD-mediated low-level resistance is mainly detectable *in vitro* and rarely identified as a consistent and reliable marker of PZA resistance in clinical setting.

## Discussion

Despite being used for more than 50 years in clinical settings, the exact mode of action of PZA remains elusive and potentially controversial with two current models biologically conflicted. In this study, we aimed at comprehensively investigate the molecular bases of these two opposite modes of action to decipher the process that underlies PZA efficacy.

By performing antimicrobial susceptibility testing at various pH, we confirmed that acidic pH drastically boosts PZA/HPOA efficacy *in vitro*. These results are in line with many seminal studies showing that PZA and HPOA are poorly active in standard Middlebrook culture media^5^ and strongly potentiated at mildly acidic pH^23, 24, 25, 62^. These findings are also in perfect agreement with more recent work showing that acidic pH is required for PZA antibacterial efficacy in more complex systems, such as infected macrophages^30, 63^ or lungs^44, 64, 65^. By using high-resolution correlative nano-secondary ion mass spectrometry imaging and scanning electron microscopy, we indeed previously demonstrated that a functional endolysosomal network displaying acidic features greatly increases PZA/HPOA enrichment accumulation in *Mtb* or its surrounding vicinity. Pharmacological or genetic modulation of *Mtb* exposure to such acidic environments had also direct impact on PZA/HPOA enrichment at the bacterial single-cell level, and ultimately a potent antibacterial activity^63^.

Capitalizing from the well-established *Mtb* pH-GFP reporter system^29, 30, 39, 42, 43^, we set up distinct *in vitro* assays to monitor non-invasively PZA/HPOA effect on *Mtb* IBpH. Across all format, both compounds lowered the IBpH of *Mtb* cytosol in a dose- and pH-dependent manner. This specific feature was also observed previously with the reference strain H37Rv during host-cell infection, where prolonged exposure to higher drug concentration IBpH acidification^30^. The use concanamycin A, a conventional v-ATPase inhibitor that impairs protons import within endolysosomes, further completely abolished PZA-mediated effect on IBpH and abrogated PZA antimicrobial activity^30, 63^. This work showed that functional endolysosomal acidification is also a critical requirement for PZA/HPOA pH-disruptive property within host-cells.

In the current study, we show that PZA/HPOA-driven IBpH acidification targets both actively growing and non-replicating *Mtb* cells demonstrating that PZA/HPOA antimicrobial activity does not rely on Mtb replication unlike other anti-TB drugs such as INH^66, 67^. This feature observed onto non-replicating cells is in accordance with previous reports from Darby *et al*. and Fontes *et al*. who relied on genetically-encoded or probe-based IBpH reporters to demonstrate that PZA/HPOA display pH-homeostasis disruptive activity in buffers^28, 29^. Moreover, our data show that even if the abundance of a given carbon source, such as glycerol, can alter PZA/HPOA efficacy, *Mtb* killing by PZA/HPOA does not require the presence of a growth permissive carbon source.

Altogether, our data support a direct correlation between extracellular protons availability, PZA/HPOA-induced IBpH collapse and growth inhibition providing strong experimental evidences for the pH-dependent PZA-mediated cytosolic acidification model originally proposed by Zhang *et al*.^24, 26^.

To test whether the marked IBpH alteration observed in PCB leads to bacterial killing, we performed time-, dose- and pH-dependent killing experiments. In PCB adjusted at pH5.5, PZA displays strong bactericidal activity. Indeed, a 14-day exposure to 50, 200 or 500 mg/L of PZA in PCB pH 5.5 led to an almost complete sterilization of the bacterial culture, whereas no killing was observed at pH 7. This result echoes previous studies showing pH-dependent killing on non-replicating bacteria at acidic pH^54^, yet differs from Peterson *et al*. who reported a two Log^10^ reduction in bacterial viability upon treatment with 200 mg/L of PZA in PBS buffer at pH 7^35^. This difference in killing efficacy at neutral pH could be due to distinct inoculum density, or alternatively the weaker buffering capacity of PBS in comparison to PCB, which could facilitate local acidification during prolonged incubation. Despite these discrepancies, the collective evidences support a model in which PZA rather acts as a slow-killing drug in growing or non-growing conditions *in vitro*; a conclusion recently reaffirmed by Gouzy *et al*. using a custom-based culture media^52^.

Our current work also shows that structurally related analogues to PZA/HPOA, display similar pH-dependent behaviour in term of IBpH homeostasis perturbation and antibacterial activity. This suggests that such mechanism is not unique to PZA/HPOA but rather conserved among structurally related molecules. Analysis of the chemical structure of this sub-class of compounds clearly supports a model in which harbouring a carboxylic acid function, or an amide readily convertible into a carboxylic acid function though enzymatic action, accounts for this phenotype, consistent with a weak acid permeation model^39, 40^.

A long-standing debate regarding the pH-dependent model of PZA/HPOA action is whether the proton shuttling effect and the resulting IBpH acidification are a direct effect of the drug or the consequence of a yet-unknown inhibited process^68^. To resolve this, we designed an *in vitro* assay, using phospholipid-based liposomes, providing a minimal system devoid of proteins or metabolic pathways. We show that HPOA, the bio-active form of the drug, is able to lower the luminal pH of these liposomes in a pH-dependent manner. The most parsimonious explanation to this process, is that HPOA alone transports protons inward, in strict accordance with the Henderson–Hasselback relationship. These findings demonstrate that HPOA-driven acidification does not depend on inhibiting any cellular process; rather, it reflects an intrinsic, pH-dependent proton-translocation mechanism.

Because compound-mediated IBpH acidification can arise by two distinct processes (*e.g*. protonophore or membrane permeant weak acid), we aimed at determining the molecular basis of this acidification process. Recent studies have suggested that the PZA/HPOA pair might act as protonophore that flips protons across the mycobacterial envelope. In this study, we show by using three independent experimental systems, the BLM, RLM and fluorescein-loaded liposomes that PZA/HPOA is not a protonophore *per se*. In contrast to well-characterized molecule CCCP, PZA/HPOA don’t repeatedly flip protons across biological membranes^68, 69, 70^. Instead, we provide strong evidence that HPOA acts as weak acid that diffuses across the membrane under its neutral protonated form, and release proton upon entry within the liposomal lumen until reaching an equilibrium. These results, together with previous observations that the acid-base equilibrium of POA^−^/HPOA is critical for efficacy^25, 28^, demonstrate that PZA/HPOA acidifies IBpH through a weak-acid permeation mechanism rather than by protonophore-mediated proton shuttling.

Finally, another key biological question addressed in our study, is the potential direct contribution of the aspartate decarboxylase PanD in PZA/HPOA efficacy. Antimicrobial susceptibility assays in standard 7H9 broth (circumneutral or acidic) showed that supplementation with Panto, strongly antagonised PZA/HPOA efficacy, as previously reported^31, 32, 41^. We also confirmed that the nature of the carbon source is a key parameter in efficacy, with glycerol having potentiating effect on this drug^38, 71^. Following this observation, we tested whether the observed antagonisms were carbon-source dependent. Intriguingly, while glycerol was replaced by oleic acid as the sole carbon source^52^, Panto-mediated antagonism was almost completely abolished. Exploiting this observation, we compared the PZA/HPOA susceptibility of the *panCD*-deleted auxotroph H37Rv mc^2^ 6206^72^ with prototrophic H37Rv parent. In the oleic acid-based medium, results highlight that the lack of functional PanCD does not affect PZA/HPOA efficacy, suggesting that PanD is unlikely to be the primary target of the drug. These data are also in accordance with the IBpH homeostasis assays that revealed that both prototrophic and auxotrophic strains are affected by PZA/HPOA-mediated acidification and that exogenous Panto supplementation did not revert this phenotype. This IBpH homeostasis alteration was also observed on non-replicating starved cells in PCB buffer in a pH-dependent manner, and time-kill experiments showed that deletion of the *panCD* genetic locus did not alter bacterial viability even after 14 days of deprivation, as previously reported^56^. On the other end, PZA treatment triggered a slow but very strong decrease in bacterial viability regardless of the presence of PanCD enzymes or the exogenous supplementation of Panto. Collectively, these data argue that PZA/HPOA act through a PanD-independent, pH-dependent protons-import mechanism.

Of note, while performing these experiments we observed some minor differences between a strain harbouring a functional PanD in comparison to a PanD-deficient strain. Indeed, in several assays the PanD-deficient strain displayed a slightly greater buffering deficit after PZA/HPOA exposure. Because PanD convert L-aspartate to β-alanine while consuming a proton, we propose that active PanD can buffer cytosolic pH under low-level drug or stress. Overexpressing PanD from *Mtb*, *M.smegmatis* or *E.coli* indeed confers low-level resistance to HPOA on standard 7H10 media, and *panD* or *clpCP* mutations that stabilise PanD were isolated in low-grade HPOA-resistant mutants (35, 39, 40). Thus, enhanced PanD activity may mitigate mild PZA/HPOA-mediated acidification without being the primary target.

Another, interesting feature that could bridge the two models is that β-alanine and Panto are known buffers that alleviate metabolic acidosis in eucaryotic cell^73, 74^. Their partial protection in *Mycobacterium* towards cytosolic acidification might arise from the same physicochemical buffering rather than pathway rescue, a hypothesis that warrants deeper investigations.

Our study underscore how profoundly experimental context shapes PZA efficacy. The artificial concentration of glycerol has long been known to modulate antibiotic susceptibility^49, 50, 71, 75, 76^. Yet, to our knowledge, it is the first instance in which a pronounced glycerol-driven antagonism, has been interpreted as evidence for an accepted anti-TB drug mode of action that appears limited to *in vitro* conditions. In our case, assays conducted in the more physiological, acidic HBO medium, revealed PZA’s full antibacterial power, highlighting its status as an unusual drug whose activity is maximised under conditions mimicking better the environment Mtb experiences during infection.

Taken together, our data support a revised model (Fig 5) in which acidic pH serves as the principal factor of PZA/HPOA efficacy against *Mtb*. In this framework, HPOA behaves as a membrane-permeant weak acid. Our findings also indicate that PanD is unlikely to be the primary target of PZA. Its decarboxylase activity, along with the buffering capacity provided by Panto, only partially counteract HPOA-driven acidification and growth inhibition, and only under certain *in-vitro* conditions. Notably, this protective effect is abolished under more physiological settings such as the acidic HBO medium, explaining why PanD-associated low-level resistance is rarely seen in clinical isolates and offers limited value as a reliable marker of PZA resistance.

## Materials and Methods

### Bacterial strains and culture conditions

Laboratory bacterial strains *Mtb* H37Ra ATCC 25177, *Mtb* H37Rv, *Mtb* H37Rv mc^2^ 6206 and *Mtb* H37Rv mc^2^ 6230 were used in this study. *Mtb* H37Rv mc^2^ 6206 and mc^2^ 6230 strains^47, 48, 72^ were kindly provided by William R. Jacobs, Jr. (Albert Einstein College of Medicine, New York, NY, USA). Bacterial cultures were routinely grown on standard Middlebrook 7H9 broth (BD Difco; #271310) supplemented with 0.2% glycerol (*v/v*) (Euromedex; #EU3550), 0.025% (*v/v*) tyloxapol (Sigma-Aldrich; #T8761) and 10% (*v/v*) oleic acid, albumin, dextrose, catalase (OADC enrichment - BD Difco; #211886). When required pantothenate (Sigma-Aldrich; #21210) was used at a final concentration of 25 mg/L to support the growth of *Mtb* H37Rv mc^2^6230 and mc^2^6206, and 50 μg/mL leucine (Sigma-Aldrich; #L8000) and 2g/L casamino acids (VWR; #J851) were also used to support the growth of *Mtb* H37Rv mc^2^6206. Recombinant *Mtb* H37Ra ATCC 25177 and *Mtb* mc^2^6230 strains expressing pH-GFP^42^ (pUV15-pHGFP; Addgene plasmid #70045, kindly gifted by Sabine Ehrt), were generated by electroporation according to Goude *et al*.^77^ and subsequently selected onto 7H11 agar medium (BD Difco; #283810) supplemented with 10% OADC and 50 mg/L Hygromycin B (Toku-E; #H007). Hygromycin B was used as selection marker for the culture maintenance of the fluorescent strain at a final concentration of 50 mg/L, but was not used during intrabacterial pH homeostasis disruption experiments.

### Antimycobacterial susceptibility testing and MIC determination

MIC determinations against *Mtb* H37Ra ATCC 25177 were performed at circumneutral and mildly acidic pH as previously described^39^. Briefly, all experiments at pH 6.8 were performed in standard Middlebrook 7H9 medium containing 0.2% glycerol and 10% OADC enrichment. To avoid Tween-80-mediated toxicity, 0.025% Tyloxapol (Sigma-Aldrich; #T8761) was used. For pH-dependent experiments, the medium was supplemented with 50 mM MES and adjusted to pH 5.5^78, 79^.

Chemical compounds including PZA (#P7136), HPOA (#P56100), NAM (#N0636), NAC (#N4126), SA (#247588), INH (#I3377), ETH (#E6005), PTH (#SMB00387), RIF (#R3501) were purchased from Sigma-Aldrich (Saint-Quentin Fallavier, France). All compounds were > 95% purity. Kanamycin (KAN) sulfate (#UK0010D) was purchased from Euromedex. Compounds were dissolved in dimethyl sulfoxide (DMSO) (DASIT group; #455103) at 50 mg/mL for PZA, HPOA, NAM, NAC, and KAN, at 20 mg/mL for SA, at 10 mg/mL for INH, at 2 mg/mL for ETH, PTH and RIF. The solutions were then sterilized using 0.22 μm syringe filters (#146560) from Clearline.

Two-fold serial dilutions of the compound stock solutions were used to obtain a range of 10–12 concentrations in the appropriate culture medium. Exponential-phase *Mtb* cultures (OD_600nm_ that approximates 0.6–1.2) were adjusted to a bacterial density of 5 × 10^6^ CFU/mL in complete 7H9 medium at pH 6.8 or pH 5.5. A volume of 100 μL of this inoculum was then dispensed into each well containing 100 μL of each serial dilution of the compound, achieving a final bacterial concentration of 2.5 × 10^6^ CFU/mL per well. Positive growth controls (*i.e*., inoculum without antibiotics or with DMSO), negative growth controls (*i.e*., 50 μg/mL KAN), and sterility controls (*i.e*., medium only) were included. The 96-well flat-bottom Nunclon Delta Surface microplates with lid (Thermo-Fisher Scientific; #167008) were incubated at 37 °C for 14–21 days for both pH conditions. The minimum inhibitory concentration (MIC) of each antibiotic at pH 6.8 or pH 5.5 was visually determined in accordance with the EUCAST and CLSI guidelines^80^. Results were confirmed by OD_600nm_ measurement using the TECAN spark 10M^™^ multimode microplate reader. The lowest antibiotic concentration that inhibited bacterial growth was determined as the MIC.

Antagonistic studies were performed in the exact same manner in the presence of Panto which was dissolved in water at 25 mg/mL and used at a final concentration of 25mg/L. For visual representation of potentiating and antagonistic effects, MIC have been expressed as Log^2^ Fold-Change (MIC) in comparison to their respective control condition. The corresponding heat-map has been generated accordingly.

Finally, when required, a half-maximal effective concentration (EC_50_) was determined using a four-parameter log-logistic nonlinear regression model (R Studio software, The R Project for Statistical Computing), as the concentration required to achieve 50% of the observed effect (i.e., *Mtb* growth inhibition). All the presented data have been obtained from experiments performed in technical triplicate at least on three distinct occasions.

### Antimycobacterial susceptibility testing in High-BSA-Oleate (HBO) media

To prepare High-BSA-Oleate (HBO) media, Middlebrook 7H9 medium was supplemented with 50g/L of fatty acid-free bovine serum albumin (BSA) (Millipore; #3117057001), 3mM oleic acid (Millipore-Sigma; OX-0165), 0.850g/L NaCl, tyloxapol 0.05% (Sigma-Aldrich; #T8761;) and 50mM of MES buffer (Sigma-Aldrich; #M3671). Glycerol was omitted. The media was acidified to pH 5.8 by the addition of concentrated HCl. A stock of PZA at 100 mg/mL in DMSO was used to distribute a gradient concentration of PZA in 384 well-plates using a HP dispenser. Cultures of mid-log phase bacteria (OD = 0.5–0.8) were washed three times in PBS and inoculated in HBO media pH5.8 to a final OD of 0.05. A volume of 50uL of bacteria were added per well. Plates were incubated for 14 days at 37°C before OD reading at 600nm. In the case of *Mtb* H37Rv mc^2^6206, the media were supplemented with Panto (25 mg/L), leucine (50 mg/L) and casamino acids (2g/L) to support bacterial growth.

### Intrabacterial pH homeostasis disruption assays

Recombinant *Mtb* ATCC 25177 and *Mtb* mc^2^6230 strains expressing the ratiometric pH-sensitive pH-GFP sensor were used to monitor IBpH homeostasis and drug-mediated perturbations on both exponentially growing and non-replicating *Mtb* cells^29, 30, 39, 42^.

*Mtb* IBpH determination on exponentially growing *Mtb* was performed as previously reported^29, 30, 39, 42^. Briefly, exponentially growing *Mtb* cultures (OD_600nm_ 0.5–1.5) were centrifuged at 3,500 rpm for 10 min. The pellet was resuspended in 10 mL of Middlebrook 7H9 medium adjusted at pH 5.5 or pH 6.8 in order to have a normalized OD_600nm_ of 0.8. Then, 100 μL of this bacterial inoculum was used to inoculate wells containing 100 μL of serially diluted compounds of interest, resulting in a final OD_600nm_ of 0.4 in each well. When required the medium was supplemented with Panto at a final concentration of 25 mg/L. After 24 h of incubation, the fluorescent pH-GFP signal was subsequently detected using excitation/emission wavelengths of λ_ex405nm_/λ_em535nm_ followed by λ_ex488nm_/λ_em535nm_ using a TECAN spark 10M^™^ multimode microplate reader (Tecan Group Ltd, France). Then fluorescence intensity ratios were calculated from data obtained at λ_em535nm_ from λ_ex405nm_/λ_ex488nm_ excitations. For experiments carried at pH 6.8, the well-established protonophore Carbonyl Cyanide *m*-Chlorophenylhydrazone (CCCP) (Sigma-Aldrich; #215911) was used as positive control of intrabacterial pH homeostasis disruption; while for experiments conducted at pH 5.5, the well-established ionophore Monensin (MON) (Sigma-Aldrich; #M5273) was used as positive control.

*Mtb* IBpH determination on non-replicating *Mtb* was performed as described above with slight modifications. Approximately, 50 mL of exponentially growing *Mtb* cultures (OD_600nm_ 0.5–1.5) were split into 5 independent 15 mL conical tubes and centrifuged at 3,500 rpm for 10 min. The pellets were washed once and re-suspended in 10 mL of PCB with pH ranging from 7 to 5.5 by increments of 0.5 to have a normalized OD_600nm_ of 0.8. Then, 100 μL of this bacterial inoculum was used to inoculate wells containing 100 μL of serially diluted compounds of interest, resulting in a final OD_600nm_ of 0.4 in each well. After 48 h of incubation, the fluorescent pH-GFP signal was recorded and expressed as fluorescent ratio as described above.

To convert pH-GFP ratios into pH units and determine *Mtb* IBpH, a calibration curve was performed. Recombinant *Mtb* H37Ra ATCC25177 expressing pH-GFP was cultivated in standard 7H9 culture medium supplemented with 10% OADC enrichment and 50 mg/L Hygromycin B until exponential phase (OD_600nm_ between 0.5–1.5). Six independent conical 15 mL-tubes containing 10 mL of bacterial culture were prepared and centrifuged at 3,500 rpm for 10 min. The supernatant was discarded, and the bacterial pellet was re-suspended in 1 mL of PCB with pH values ranging from 8 to 5.5 in 0.5-unit increments. Following a 2-hours incubation at 37 °C, the bacterial cells were mixed with 200 μL of 0.1 mm diameter glass beads (BioSpec), and disrupted during 3 × 4 min of violent shaking using Mini-Beadbeater-96 (BioSpec, Bartlesville, OK, USA). The lysates were centrifuged for 5 min at 1,500 rpm, the resulting supernatant (~750 μL) was carefully collected, and 3 × 200 μL were transferred into each well of a 96-well plate (Nunclon Delta Surface microplate). pH-GFP signal was detected using excitation/emission wavelengths of λ_ex405nm_/λ_em535nm_ followed by λ_ex488nm_/λ_em535nm_ using the TECAN spark 10M^™^ multimode microplate reader. Fluorescent ratios were used to build a non-parametric calibration curve, by applying the locally estimated scatterplot smoothing (LOESS) prediction model in R (R Studio software, The R Project for Statistical Computing, version 1.3.1073) that fits the obtained data to a smooth standard curve. This LOESS prediction model was then used to correlate fluorescence ratio with their respective IBpH values. All the results were exported as CSV files, imported into the R Studio software, and graphical representations were plotted using the ggplot2 package (version 3.5.0). Results of all the presented data are have been performed in technical triplicate at least on three distinct occasions.

### PZA bactericidal activity on non-replicating *Mtb* using spot CFU-based assays

Exponentially growing cultures of *Mtb* H37Ra pH-GFP and *Mtb* mc^2^ 6230 pH-GFP were centrifuged, the supernatant was removed, the pellets were washed and resuspended in PCB adjusted to pH 7 or pH 5.5, and supplemented with 50mg/L hygromycin B to obtain a final OD_600nm_ of 0.1. PZA was used at final concentration of 0, 50, 200 or 500 mg/L. MON or RIF compound was used as internal positive growth inhibition control. When required, 25 mg/L of Panto was also added to the medium. At each time point (day 0, 2, 7 and 14), 200 μL of the undiluted condition (10^0^) was sampled. From this condition, serial dilutions were carried out to obtain the dilution ranging from 10^−1^ to 10^−5^. Then, 10 μL of each dilution were spotted onto a 7H10 agar plate supplemented with 10% OADC, 50 mg/L Hygromycin B and 25 mg/L Panto to recover *Mtb* cells. The plates were incubated 21 days at 37 °C. Scanning of the plates was performed using a ChemiDoc^™^ MP Imaging System (Bio-Rad) where bacterial spots imaging was achieved by using the ‘White Epi Illumination’ setting combined with the ‘Standard Filter’. Experiments were performed on two independent occasions and are representative of one replicate.

### Isolation of rat liver mitochondria

Rat liver mitochondria were isolated by differential centrifugation^81^ in a medium containing 250 mM sucrose, 5 mM MOPS, 1 mM EGTA, pH 7.4. The final washing was performed in the medium containing bovine serum albumin at final concentration 0.1 mg/mL. Protein concentration was quantified using the Biuret method. All animal handling and experimental procedures were conducted in accordance to the international guidelines for animal care and use and received approval from the Institutional Ethics Committee of the A.N. Belozersky Institute of Physico-Chemical Biology at the Moscow State University (Protocol #3 on February 12, 2018).

### Mitochondria respiration

Respiration of isolated mitochondria was measured using a standard polarographic technique with a Clark-type oxygen electrode (Strathkelvin Instruments, UK) at 25°C using the 782 Oxygen System software. The incubation medium contained 250 mM sucrose, 5 mM MOPS, and 1 mM EGTA, pH 7.4. The mitochondrial protein concentration was 0.8 mg/mL. The reported oxygen uptake values are expressed as nmol/min/mg of protein.

### Membrane potential measurement in isolated mitochondria

Mitochondrial membrane potential was estimated using safranine O dye^82^. Fluorescence was monitored with a Panorama Fluorat 02 spectrofluorimeter (Lumex, Saint-Petersburg) at λ_ex520nm_/λ_em580nm_. At the end of each measurement, 200 nM CCCP was introduced to collapse the membrane potential. The incubation medium consisted of 250 mM sucrose, 5 mM MOPS, 0.5 mM KH_2_PO_4_, 1 mM EGTA, 2 μM rotenone, 5 mM succinate (pH 7.4), 1 μg/mL oligomycin, 15 μM safranine O. The mitochondrial protein content ranged from 0.6 to 0.9 mg protein/mL and the temperature was maintained constant at 26 °C.

### Planar bilayers

Bilayer lipid membrane (BLM) was created using the brush technique^83^ from a 2% decane solution of phosphatidylcholine derivative, diphytanoylphosphatidylcholine (DPhPC) (Avanti Polar Lipids; Alabaster, AL). on a 0.6-mm aperture in a Teflon septum separating the experimental chamber into two compartments of equal 3-ml volume. The electrical current across the BLM was measured under voltage-clamp conditions with two AgCl electrodes placed into the solutions on the two sides of the BLM through agar bridges, using a Keithley 428 amplifier (Cleveland, Ohio, USA). The voltage applied to the BLM was set at 50 mV.

### Pyranine-loaded liposomes

The pH of liposomes was measured as previously described by Chen *et al*.^58^ with slight modifications. To prepare pyranine-loaded liposomes, a lipid mixture from POPC, POPG and cholesterol (5.4, 1.5 and 3.1 mg, respectively) dissolved in a chloroform suspension was dried in a round-bottom flask under a nitrogen stream. The lipid was then resuspended in 1 mL buffer (100 mM KCl, 20 mM MES, 20 mM MOPS, 20 mM Tricine titrated with KOH to pH 8.0) containing 0.5 mM pyranine. The suspension was vortexed and freeze-thawed three times. Unilamellar liposomes were then prepared by extrusion through 0.1 μm pores (Nucleopore polycarbonate membranes) using an Avanti Mini-Extruder. Untrapped pyranine was removed by passage through a Sephadex G-50 coarse column equilibrated with the same buffer solution. The liposomes were diluted in the same buffer at pH 6.0 and final lipid concentration in the cuvette of approximately 20 μg/mL. To measure the pH dissipation rate in liposomes, the suspension was supplemented with 2 mM *p*-xylene-*bis*-pyridinium bromide to quench the fluorescence of leaked pyranine. The internal pH was estimated by measuring the fluorescence intensity at λ_ex455nm_/λ_em505nm_, as the fluorescence emission of liposome-loaded pyranine monotonously increases upon excitation at λ_ex455nm_ over a pH range of 6.0 to 7.9^58^. The fluorescence was monitored on a Panorama Fluorat 02 spectrofluorimeter (Lumex, Saint-Petersburg)^84^. At the end of each recording, 1 μM X537A (Lasalocid A) was added to dissipate the remaining pH gradient. In several experiments, to prevent the formation of H^+^-diffusion potential, the experiments were conducted in the presence of 10 nM K^+^-carrier valinomycin. To minimize the leakage of H^+^ from liposomes, the experiments were performed at 15°C.

### Quantification and statistical analysis

All results displayed in this study were obtained from biologically independent experiments (*n* = 3) each performed in at least three technical replicates unless otherwise stated. Statistical analysis for IBpH homeostasis disruption assays was performed comparing the means between the conditions of interest using one-way ANOVA followed by Tukey’s post-test with the ‘aov()’ and ‘TukeyHSD()’ functions in R Studio using the ggpubr R package (The R Project for Statistical Computing). All *p*-values presented in the text or figures are relative to the control condition or to the lowest drug concentration applied in dose-response experiments. A *p*-value was considered significant if *p-value* < 0.05. Statistical analysis is displayed in figures as follows: n.s., not significant; *, *p*-value < 0.05; **, *p*-value < 0.01; ***, *p*-value < 0.001. Each figure or table legend specifies the statistical tests used, the number of biologically independent replicates and the number of technical replicates.

## Supplementary Material

1

## Abbreviations:

PZA: Pyrazinamide
TB: Tuberculosis
*Mtb*: *Mycobacterium tuberculosis*
HPOA: Pyrazinoic acid
POA^−^: Pyrazinoate anion
INH: Isoniazid
RIF: Rifampicin
EMB: Ethambutol
NAD+: Nicotinamide adenine dinucleotide
FAS I: Fatty acid synthase I
CoA: Coenzyme A
MIC: Minimal inhibitory concentration
NAM: Nicotinamide
NAC: Nicotinic acid
SA: Salicylic acid
ETH: Ethionamide
PTH: Prothionamide
β-ala: β-alanine
Panto: Pantothenate
MON: Monensin
CCCP: carbonyl cyanide *m*-chlorophenyl hydrazone
IBpH: Intrabacterial pH
LOESS: Locally estimated scatterplot smoothing
EC50: Half maximal effective concentration
HBO: High-BSA-Oleate
PCB: Phosphate-citrate buffer
BLM: Bilayer lipid membrane
DNP: 2,4-dinitrophenol
