# Supplementary material for: Pyrazinamide kills Mycobacterium tuberculosis via pH-driven weak-acid permeation and cytosolic acidification
Source: bioRxiv. 2025 Sep 27:2025.09.26.678883. Preprint. [Version 1] doi: 10.1101/2025.09.26.678883 (PMC12486132; doi:10.1101/2025.09.26.678883)
Supplement: 1 [file NIHPP2025.09.26.678883V1-supplement-1.pdf]

bioRxiv preprint doi: https://doi.org/10.1101/2025.09.26.678883; this version posted September 27, 2025. The copyright holder for this preprint (which was not certified by peer review) is the author/funder, who has granted bioRxiv a license to display the preprint in perpetuity. It is made available under aCC-BY 4.0 International license.

Supporting information

Pyrazinamide kills *Mycobacterium tuberculosis* via pH-driven weak-acid permeation and cytosolic acidification

Janïs Laudouze[1], Tatyana I. Rokitskaya[2], Akira Abolet[1], Vanessa Point[1], Alexander M. Firsov[2], Ljudmila S. Khailova[2], Jean-François Cavalier[1], Stéphane Canaan[1], Alain R. Baulard[3], Yuri N. Antonenko[2]*, Alexandre Gouzy[4]* & Pierre Santucci[1]*&

[1] Aix Marseille Univ, CNRS, LISM, IMM FR3479, IM2B, Marseille, France

[2] Belozersky Institute of Physico-Chemical Biology, Lomonosov Moscow State University, 119991 Moscow, Russia

[3] Univ. Lille, CNRS, Inserm, CHU Lille, Institut Pasteur de Lille, U1019 - UMR9017 - CIIL - Center for Infection and Immunity of Lille, F-59000 Lille, France.

[4] Department of Microbiology and Immunology, Weill Cornell Medical College, New York, New York, USA.

* Correspondence address to Yuri N. Antonenko, antonen@belozersky.msu.ru

* Correspondence address to Alexandre Gouzy, alg2053@med.cornell.edu

* Correspondence address to Pierre Santucci, psantucci@imm.cnrs.fr

[&] Lead contact

Short title: Pyrazinamide bactericidal mode of action

Keywords: acidic environments, acid-base equilibrium, intrabacterial pH homeostasis, protonophore, pantothenate, glycerol

---

Supplementary Fig1 to Supplementary Fig9 with their legends.

bioRxiv preprint doi: https://doi.org/10.1101/2025.09.26.678883; this version posted September 27, 2025. The copyright holder for this preprint (which was not certified by peer review) is the author/funder, who has granted bioRxiv a license to display the preprint in perpetuity. It is made available under aCC-BY 4.0 International license.

Figure S1

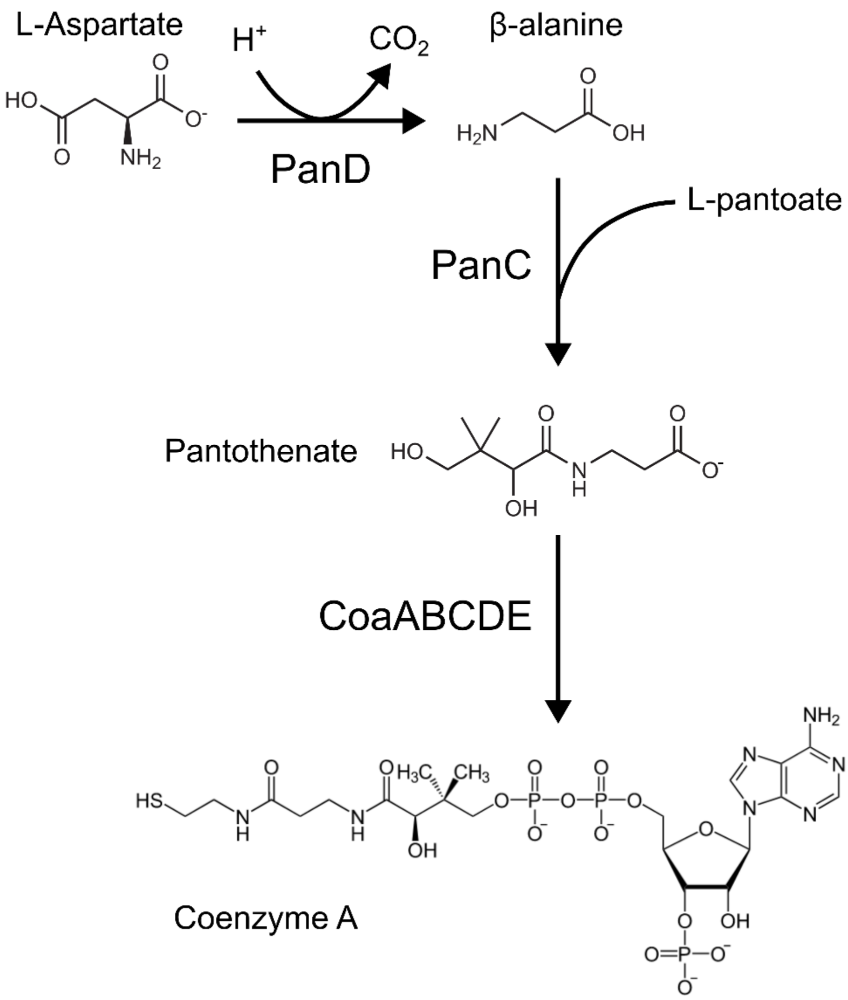

1203

1204 **Figure S1. Schematic representation of CoA biosynthesis involving PanCD and**
1205 **CoaABCDE enzymes.**

bioRxiv preprint doi: https://doi.org/10.1101/2025.09.26.678883; this version posted September 27, 2025. The copyright holder for this preprint (which was not certified by peer review) is the author/funder, who has granted bioRxiv a license to display the preprint in perpetuity. It is made available under aCC-BY 4.0 International license.

Figure S2

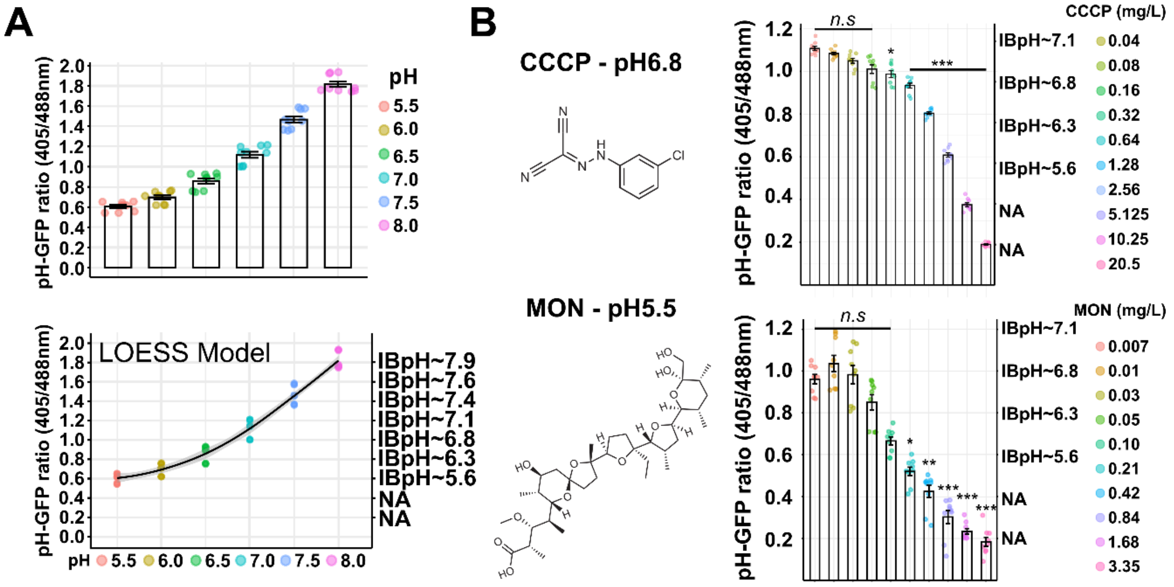

Figure S2. *in vitro* monitoring of *Mtb* IBpH levels and assessment of compound-mediated disruption at both circumneutral and acidic pH. (A) Determination of a calibration curve of *Mtb* H37Ra IBpH homeostasis. *Mtb* pH-GFP cell lysate was resuspended in PCB with pH values adjusted between pH 5.5 and pH 8. After 48 hours, fluorescence intensity at λ_{em} 535nm recorded after excitation at λ_{ex} 405nm and λ_{ex} 488nm. pH-GFP fluorescence ratios were determined (*top* panel) and used to build a LOESS model that correlates fluorescence intensities with pH values (*bottom* panel). Each measurement is the average of 3 technical replicates performed on 3 distinct occasions. **(B)** Quantification of CCCP and Monesin-induced *Mtb* H37Ra IBpH alteration at circum-neutral and acidic pH. *Mtb* pH-GFP ratio were determined in the presence of increasing concentrations of the protonophore CCCP or the pH-dependent ionophore MON after 24 h exposure at pH 6.8 or pH 5.5 respectively. *Mtb* IBpH results displayed in this figure were obtained from 3 biologically independent experiments and are displayed as mean ± SEM. In dose response analysis, statistical significance was assessed by comparing the means of each concentration with the lowest concentration tested using one-way ANOVA followed with Tukey's multiple comparisons test. All *p*-values were considered significant when *p*-value < 0.05.

bioRxiv preprint doi: https://doi.org/10.1101/2025.09.26.678883; this version posted September 27, 2025. The copyright holder for this preprint (which was not certified by peer review) is the author/funder, who has granted bioRxiv a license to display the preprint in perpetuity. It is made available under aCC-BY 4.0 International license.

Figure S3

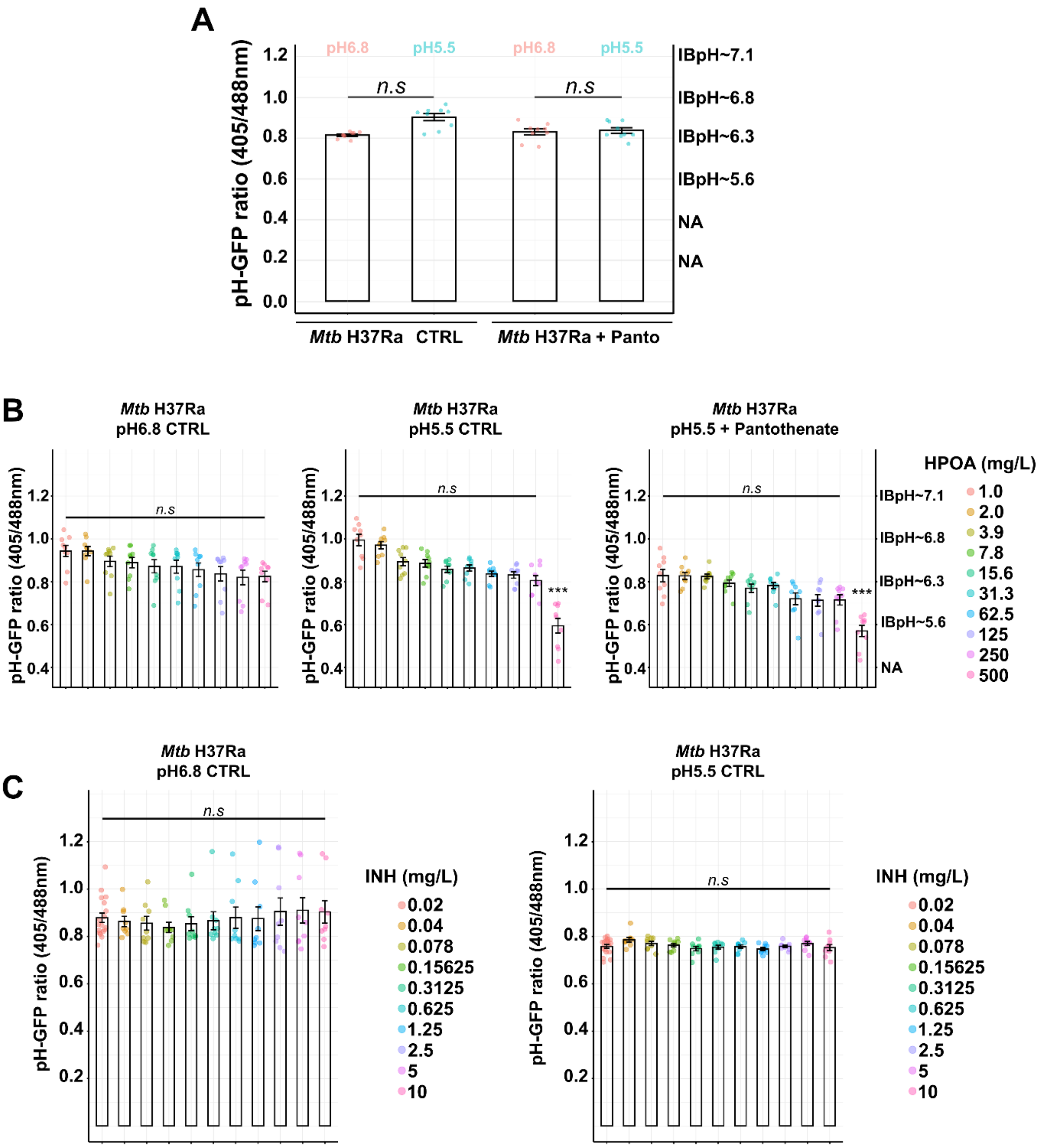

Figure S3. Analysis of *Mtb* IBpH homeostasis maintenance in replicating cells following incubation at circumneutral and acidic pH, in the presence of Panto or in the presence of control drugs. (A) The IBpH of replicating *Mtb* is not significantly affected by external pH or Panto levels. Quantification of *Mtb* H37Ra IBpH alteration were performed at circum-neutral or acidic pH in the absence (*left* panel) or presence (*right* panel) of 25 mg/L of Panto. Results were obtained after 24 h of incubation in each condition. **(B)** Quantification of HPOA-induced *Mtb* H37Ra IBpH alteration at circum-neutral (*left* panel), acidic pH (*middle* panel) and acidic pH in the presence of 25 mg/L of Panto (*right* panel). *Mtb* pH-GFP ratio were determined in

bioRxiv preprint doi: https://doi.org/10.1101/2025.09.26.678883; this version posted September 27, 2025. The copyright holder for this preprint (which was not certified by peer review) is the author/funder, who has granted bioRxiv a license to display the preprint in perpetuity. It is made available under aCC-BY 4.0 International license.

the presence of increasing concentrations of HPOA after 24 h of exposure. **(C)** Quantification of INH-induced *Mtb* H37Ra IBpH alteration at circum-neutral (*left* panel) and acidic pH (*right* panel). *Mtb* pH-GFP ratio were determined in the presence of increasing concentrations of control drugs after 24 h of exposure. *Mtb* IBpH results were obtained from 3 biologically independent experiments and are displayed as mean ± SEM. In dose response analysis, statistical significance was assessed by comparing the means of each concentration with the lowest concentration tested using one-way ANOVA followed with Tukey's multiple comparisons test. All *p*-values were considered significant when *p*-value < 0.05.

bioRxiv preprint doi: https://doi.org/10.1101/2025.09.26.678883; this version posted September 27, 2025. The copyright holder for this preprint (which was not certified by peer review) is the author/funder, who has granted bioRxiv a license to display the preprint in perpetuity. It is made available under aCC-BY 4.0 International license.

Figure S4

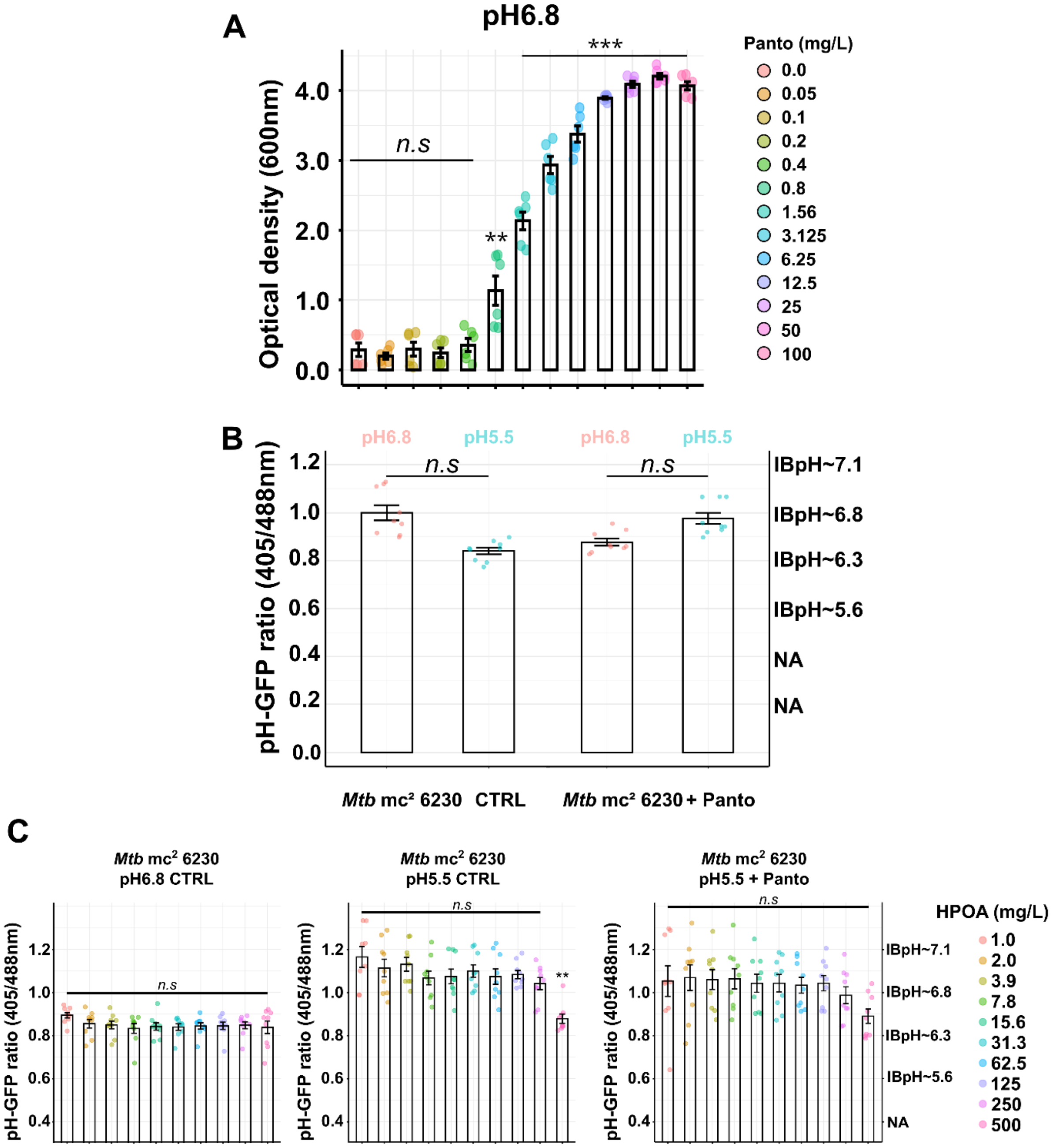

Figure S4. Analysis of *Mtb* mc^2 6230 auxotrophy and IBpH homeostasis maintenance in standard 7H9 media following incubation at circumneutral and acidic pH. (A) Pantothenate auxotrophy experiments confirming the non-growing phenotype of *Mtb* mc^2 6230 in the absence of exogenous Panto. A dose response analysis of Panto supplementation was performed confirming that concentrations greater than 6.25 mg/L support replication, with optimal growth observed when using 25 mg/L and beyond. **(B)** The IBpH of *Mtb* mc^2 6230 is not significantly affected by external pH or Panto levels. Quantification of *Mtb* mc^2 6230 IBpH alteration were performed at circum-neutral or acidic pH in the absence (*left* panel) or presence

bioRxiv preprint doi: https://doi.org/10.1101/2025.09.26.678883; this version posted September 27, 2025. The copyright holder for this preprint (which was not certified by peer review) is the author/funder, who has granted bioRxiv a license to display the preprint in perpetuity. It is made available under aCC-BY 4.0 International license.

(*right* panel) of 25 mg/L of Panto. Results were obtained after 24 h of incubation in each condition. **(C)** Quantification of HPOA-induced *Mtb* H37Ra IBpH alteration at circum-neutral (*left* panel), acidic pH (*middle* panel) and acidic pH in the presence of 25 mg/L of Panto (*right* panel). *Mtb* pH-GFP ratio were determined in the presence of increasing concentrations of HPOA after 24 h of exposure. *Mtb* IBpH results were obtained from 3 biologically independent experiments and are displayed as mean ± SEM. In dose response analysis, statistical significance was assessed by comparing the means of each concentration with the lowest concentration tested using one-way ANOVA followed with Tukey's multiple comparisons test. All *p*-values were considered significant when *p*-value < 0.05.

bioRxiv preprint doi: https://doi.org/10.1101/2025.09.26.678883; this version posted September 27, 2025. The copyright holder for this preprint (which was not certified by peer review) is the author/funder, who has granted bioRxiv a license to display the preprint in perpetuity. It is made available under aCC-BY 4.0 International license.

Figure S5

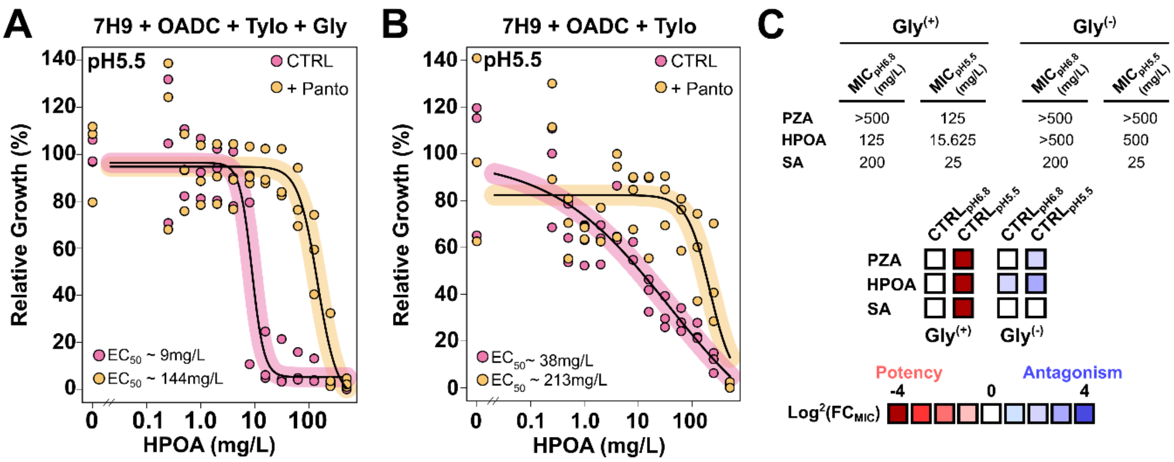

Figure S5. PZA/HPOA efficacy and Panto-mediated antagonism are impacted by carbon sources. (A-B) Comparative antibiotic susceptibility of *Mtb* H37Ra towards HPOA performed at mildly acidic pH in 7H9+OADC+Tyloxapol culture media that contains (**A** – *left* panel) or not 0.2% glycerol (**B** – *right* panel). Experiments were performed in the absence (pink) or presence of Panto (yellow) antagonist. Results are expressed as relative growth % where the no drug condition represents 100%. Dose-response curves displayed were obtained following a 4-parameter nonlinear logistic regression and EC_{50} were determined accordingly. **(C)** Summary of glycerol effect on PZA/HPOA efficacy and antagonisms. Results are expressed as minimal inhibitory concentration (MIC) and further displayed as color-coded heatmap from red (potency) to blue (antagonism). Heatmap intensities for each compound represent the fold change (FC) in MIC values expressed as $Log^2(FC_{MIC})$ by applying the following formula $MIC_{pH5.5}/MIC_{pH6.8}$ or $MIC_{Antagonist}/MIC_{CTRL}$. Results are representative of three independent replicates performed on three distinct occasions.

bioRxiv preprint doi: https://doi.org/10.1101/2025.09.26.678883; this version posted September 27, 2025. The copyright holder for this preprint (which was not certified by peer review) is the author/funder, who has granted bioRxiv a license to display the preprint in perpetuity. It is made available under aCC-BY 4.0 International license.

Figure S6

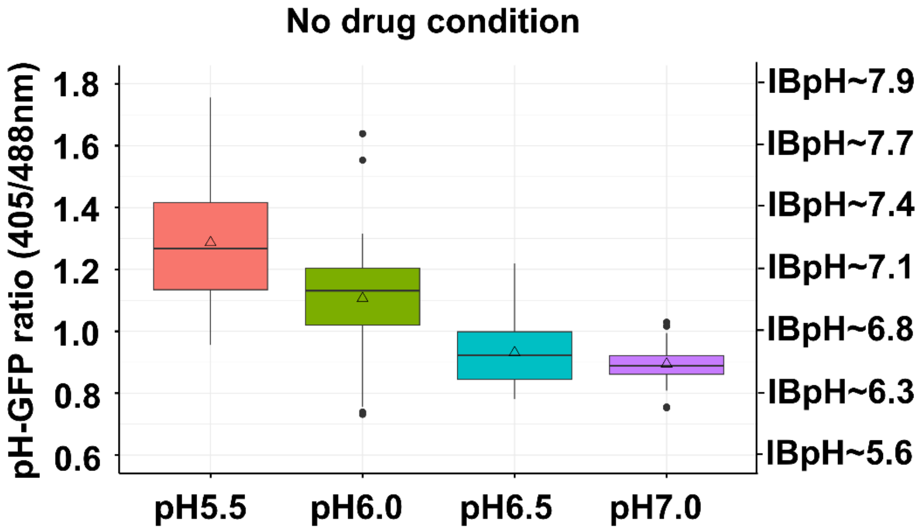

Figure S6. Analysis of *Mtb* IBpH homeostasis maintenance in non-replicating starved cells following incubation in PCB at different pH. (A) pH-dependent analysis of *Mtb* H37Ra IBpH homeostasis. pH-GFP ratios were determined after a 48 h incubation period in PCB buffer adjusted at pH 7 (purple), pH 6.5 (blue), pH 6 (green) and pH 5.5 (red).

bioRxiv preprint doi: https://doi.org/10.1101/2025.09.26.678883; this version posted September 27, 2025. The copyright holder for this preprint (which was not certified by peer review) is the author/funder, who has granted bioRxiv a license to display the preprint in perpetuity. It is made available under aCC-BY 4.0 International license.

Figure S7

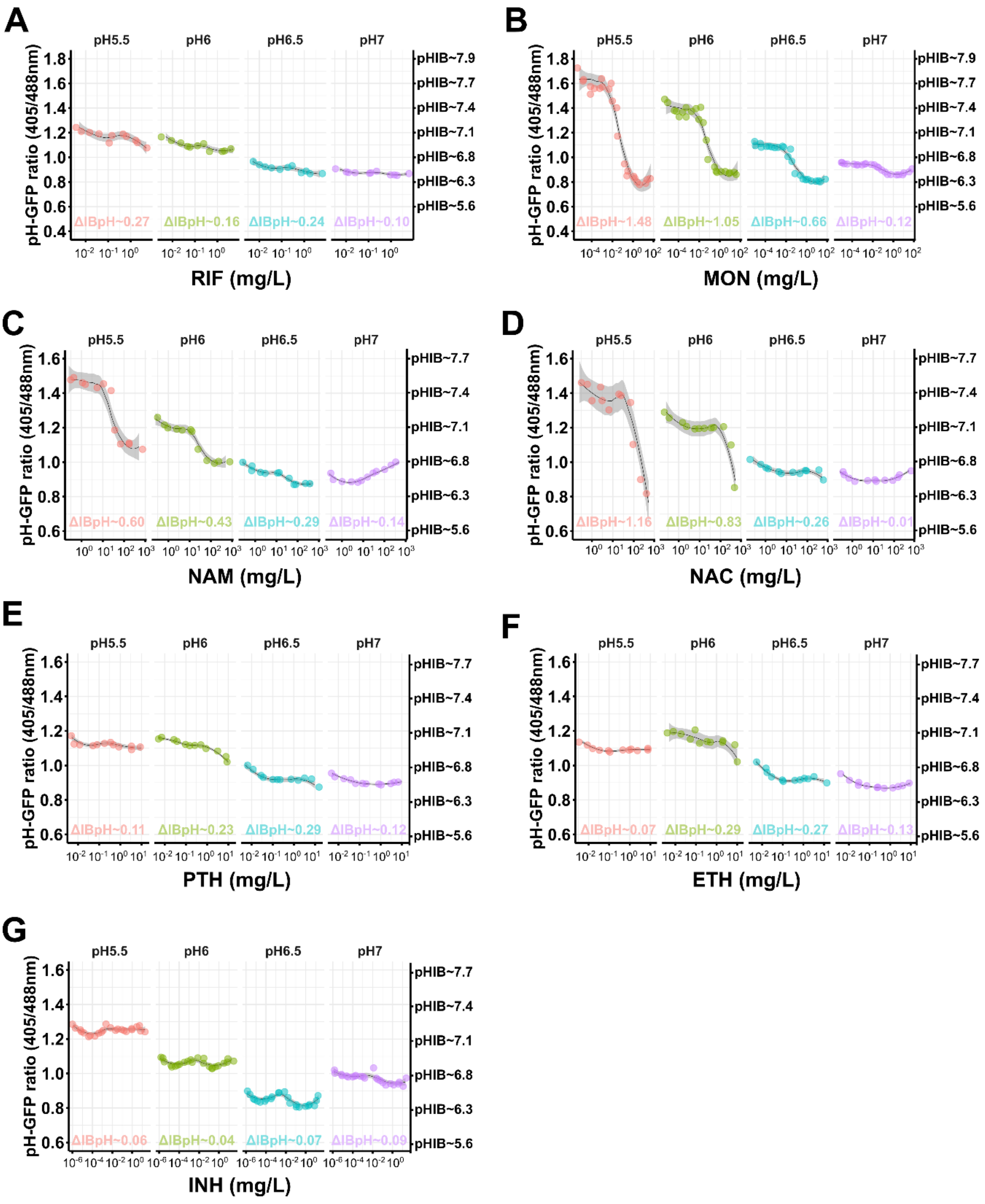

Figure S7. Analysis of *Mtb* IBpH homeostasis in non-replicating cells upon treatment with a large panel of drugs. (A-G) pH-dependent effect of RIF (**A**), and MON (**B**), NAM (**C**), NAC (**D**), PTH (**E**), ETH (**F**) and INH (**G**) on *Mtb* H37Ra IBpH homeostasis at distinct pH. Dose-response analysis were performed in PCB buffer adjusted at pH 7 (purple), pH 6.5 (blue), pH 6 (green) and pH 5.5 (red). *Mtb* were exposed to increasing concentrations of RIF or MON for 48 h before performing pH-GFP ratio recording and determination.

bioRxiv preprint doi: https://doi.org/10.1101/2025.09.26.678883; this version posted September 27, 2025. The copyright holder for this preprint (which was not certified by peer review) is the author/funder, who has granted bioRxiv a license to display the preprint in perpetuity. It is made available under aCC-BY 4.0 International license.

Figure S8

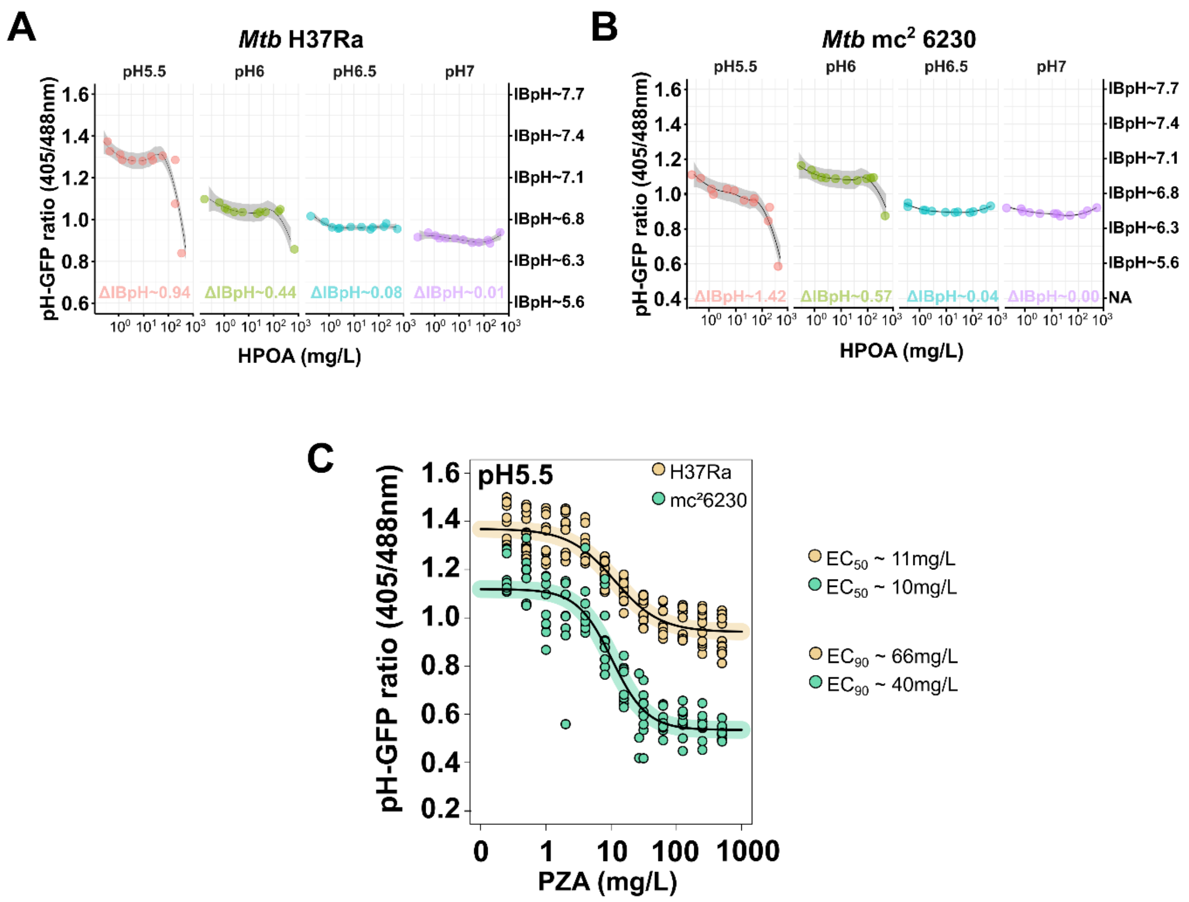

Figure S8. Analysis of *Mtb* IBpH homeostasis in non-replicating cells upon treatment with PZA/HPOA. (A-B) pH-dependent PZA-mediated IBpH cytosolic acidification of *Mtb* H37Ra (**A** – *left* panel) and *Mtb* mc^2 6230 (**B** – *right* panel). Dose-response analysis were performed in PCB buffer adjusted at pH 7 (purple), pH 6.5 (blue), pH 6 (green) and pH 5.5 (red). *Mtb* were exposed to increasing concentrations of HPOA from 0.25 to 500 mg/L for 48 h before pH-GFP ratio recording. Using pH-GFP ratios, distinct models were built using the LOESS function and ΔIBpH were calculated by comparing the IBpH recorded at the highest HPOA concentration tested with the one at the lowest HPOA concentration included in the dose response for each tested pH. **(C)** Comparative analysis of PZA-mediated IBpH alteration against *Mtb* H37Ra and mc^2 6230 in PCB buffer at pH5.5. Dose-response analysis displayed were obtained following a 4-parameter nonlinear logistic regression of the data displayed in **Fig 3A** and **Fig 3B**, and EC_{50} and EC_{90} were determined accordingly.

bioRxiv preprint doi: https://doi.org/10.1101/2025.09.26.678883; this version posted September 27, 2025. The copyright holder for this preprint (which was not certified by peer review) is the author/funder, who has granted bioRxiv a license to display the preprint in perpetuity. It is made available under aCC-BY 4.0 International license.

Figure S9

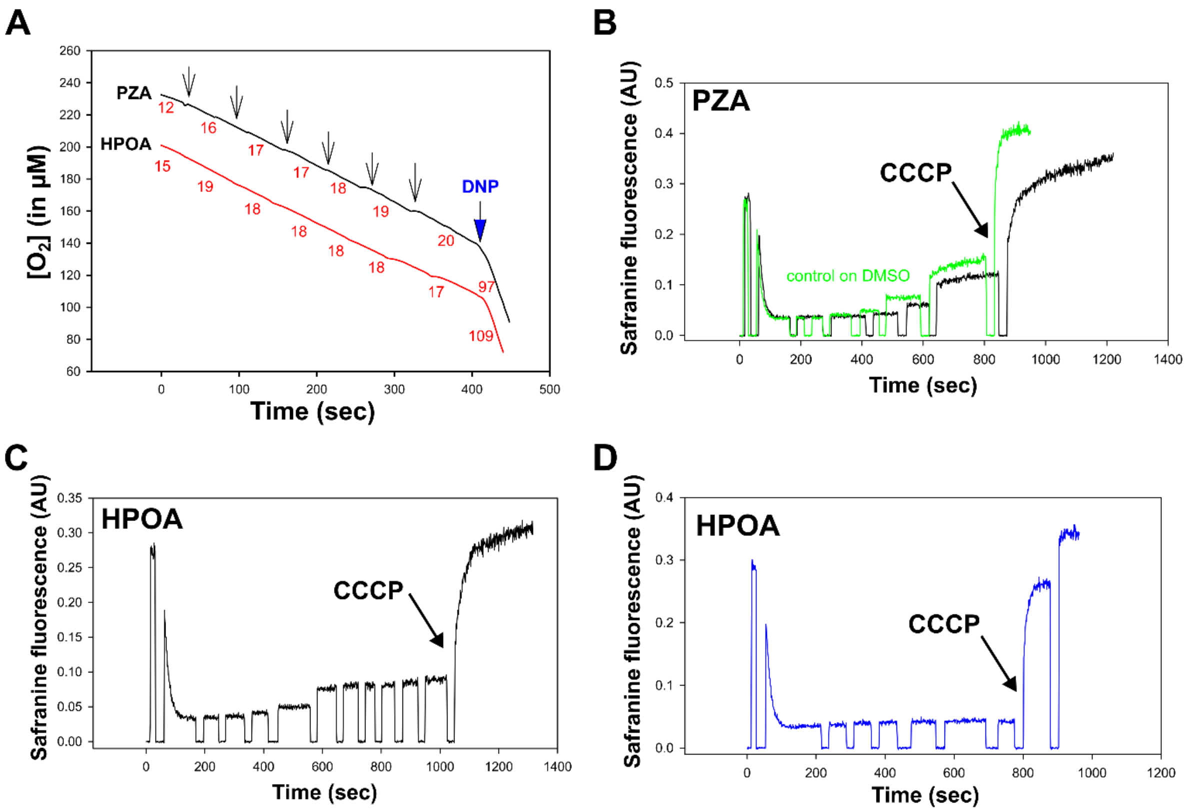

Figure S9. PZA/HPOA has no effect on mitochondrial respiration and membrane potential. (A) Effect of PZA and HPOA on respiration of rat liver mitochondria (RLM). The substrate was succinate supplemented with 2 µM rotenone. Incubation medium contained 250 mM sucrose, 20 mM MOPS, 1 mM KH_2PO_4, 2 mM $MgCl_2$, 1 mM EGTA, pH 7.2, and mitochondria (0.5 mg protein/mL). Numbers at different parts of each record are respiration rates in relative units. At the ned of the kinetic, 40 µM DNP was added as positive control. **(B-C-D)** Effect of PZA (**B**) and HPOA (**C-D**) on mitochondrial membrane potential estimated with the fluorescence of potential-sensitive dye safranine O (15 µM) in isolated rat liver mitochondria (RLM). Y-axis shows fluorescence of safranine at 580 nm (λ_{ext} 520 nm). Increasing volume 2 µL, 4 µL, 10 µL, 20 µL and 40 µL of PZA (100 mg/mL), HPOA (7 mg/mL) or DMSO were added to sample and changes in safranine O fluorescence were recorded. The substrate was succinate supplemented with 2 µM rotenone. 200 nM CCCP was added as positive control at the end of the kinetics.
